# A Low-Carbon, Recycled Powder-Based Binder: Engineering Properties, Carbon Sequestration Potential, and Recycling

**DOI:** 10.3390/ma19183855

**Published:** 2026-09-10

**Authors:** Junkai Pan, Yu Zhang, Xuexia Wang, Zhaolin Yao, Yao Ran, Renjuan Sun, Yanhua Guan, Linglai Bu

**Affiliations:** 1 Shandong Dongqing Highway Co., Ltd., Dongying 257000, China; 2 School of Qilu Transportation, Shandong University, Jinan 250002, China

**Keywords:** recycled concrete powder, recycled powder-based binder, wet carbonation, CO_2_ sequestration, post-carbonation activity, multi-objective optimization

## Abstract

**Highlights:**

• Matrix analysis identified a balanced LCRPB formulation with multiple performance objectives.
• XRD identified CaCO_3_ as the main carbonation product with vaterite in low-OPC mixtures.
• Excessive RCP reduced CO_2_ sequestration capacity because of dominant dilution effects.
• Wet carbonation was governed by alkaline leaching, pH evolution, and carbonate precipitation.

**Abstract:**

To improve the high-value utilization of recycled concrete powder (RCP) and its carbon sequestration potential, this study developed a low-carbon recycled powder-based binder (LCRPB) incorporating RCP, silica fume (SF), granulated blast-furnace slag (GBFS), and fly ash (FA). A five-factor, five-level orthogonal design was used to evaluate workability, mechanical properties, hydration behavior, wet carbonation performance, and post-carbonation reusability. The water-to-binder ratio showed the greatest relative influence on flowability and compressive strength, whereas RCP content had a greater influence on 28-day flexural strength and wet carbonation behavior. At favorable dosage levels, 2% SF, 7.5% GBFS, and 10% FA increased the 28-day compressive strength by 17.43%, 12.43%, and 11.83%, respectively. Wet carbonation showed the highest efficiency at a liquid-to-solid ratio of 5 and a CO_2_ flow rate of 3 L/min, with CaCO_3_ identified as the main carbonation product. The carbonated powder retained reuse potential. Matrix analysis identified a balanced formulation of W/B = 0.40, 0% SF, 7.5% GBFS, 10% FA, and 10% RCP. The selected formulation reduced the material-related carbon footprint by 26.6% relative to the 100% OPC reference.

## 1. Introduction

Against the backdrop of the “dual carbon” goals, the building materials industry faces the dual challenge of reducing carbon emissions and increasing the recycling rate of construction and demolition waste. As the primary binder in concrete, cement production involves significant energy consumption and CO_2_ emissions. According to statistics, CO_2_ emissions from the cement industry account for approximately 7–8% [1] of total global anthropogenic carbon emissions, making it a key target for carbon reduction in the construction sector. Therefore, reducing clinker consumption, increasing the utilization rate of industrial and construction solid waste, and developing low-carbon cementitious materials have become crucial pathways for achieving a green and low-carbon transition in the construction industry.

As urban renewal and infrastructure demolition projects continue to expand, waste concrete has become a major source of construction solid waste. After sorting and crushing, waste concrete can be processed into recycled coarse aggregate, recycled fine aggregate, and RCP. Particles with a particle size of less than 150 μm are generally considered RCP, which accounts for 20–30% of the total volume of waste concrete. Some studies have also produced RCP by further grinding recycled aggregates [2,3]. Compared to recycled aggregates, RCP has a lower resource utilization rate [4]; large stockpiles not only occupy land resources but may also cause environmental pollution. Therefore, achieving high-value utilization of RCP is of great significance for promoting the comprehensive resource utilization of all components of waste concrete. Existing research indicates that RCP contains some unhydrated cement particles and certain amounts of components such as SiO_2_, Al_2_O_3_, and CaO; therefore, it possesses potential hydraulic and pozzolanic activity [5]. Some scholars have pointed out that RCP exhibits relatively low reactivity when used as an auxiliary cementitious material [4], while Gupta et al. and Xiao et al. believe it is more suitable as a filler than as a mineral admixture [6,7]. However, further mechanical grinding can improve the particle size distribution of RCP and expose its unhydrated particles, thereby enhancing its reactivity [8,9]. Some of these unhydrated CaO particles can promote secondary hydration and accelerate C–S–H gel formation [4]. Therefore, RCP still holds significant application potential in low-carbon cementitious material systems.

To date, the effects of RCP on the workability and mechanical properties of cementitious materials have been studied to some extent. Due to the irregular particle morphology and large specific surface area of RCP, its incorporation typically reduces the flowability of the slurry and increases the water demand of the system [10,11]. Karatas et al. pointed out that although RCP reduces the flowability of the slurry, it can increase the slurry’s viscosity and cohesion [12]; Duan et al. [13] found that when the RCP content was 20%, the yield stress of the slurry could reach 3.35–5.88 times that of the unmodified system. In terms of mechanical properties, as the replacement rate of RCP increases, the compressive strength of the system typically decreases due to the dilution effect [14]. However, an appropriate amount of RCP may also improve the pore structure and promote hydration reactions through filling effects, nucleation effects, and internal curing, thereby enhancing concrete strength [7,15]. For example, Xiao et al. [7] found that when the RCP content was 15%, the compressive strength of the concrete increased by 5.9% compared to the control group. Furthermore, the porous structure of RCP can adsorb free water and gradually release it over time, thereby promoting sustained hydration reactions [16]. However, when the replacement rate exceeds 30%, the concrete strength is typically significantly lower than that of ordinary concrete due to a marked reduction in effective cementitious components [17,18,19,20]. Therefore, in current engineering applications, RCP is still predominantly used at low dosages as a direct substitute for cement. These findings indicate that the mechanical performance of RCP-containing cementitious materials should be interpreted in conjunction with the underlying hydration and microstructural characteristics rather than from strength measurements alone. Bica et al. [21] combined mechanical testing with microstructural characterization of modified concrete and demonstrated the importance of relating macroscopic mechanical behavior to changes in hydration and microstructural development.

In recent years, the combined use of different mineral admixtures in composite cementitious systems has attracted increasing attention as a strategy to reduce cement consumption and improve material performance. Mineral admixtures such as silica fume (SF), granulated blast-furnace slag (GBFS), and fly ash (FA) possess distinct chemical compositions and reaction characteristics, allowing them to improve system performance through pozzolanic reactions, latent hydraulic reactions, and micro-filling effects. SF exhibits high pozzolanic activity and micro-filling capacity and can improve matrix compactness, whereas GBFS possesses latent hydraulic activity under alkaline conditions. Rocha et al. [16] investigated the hydration characteristics of recycled powder–cement binary and multicomponent systems and found that GBFS contributed to medium- and later-age performance development. FA can also improve long-term performance and reduce material costs. Eckert et al. [22] evaluated the performance and carbon emissions of recycled fine powder-based cementitious materials and demonstrated the potential contribution of FA to reducing cement-related carbon emissions. Previous studies have suggested that the combined use of RCP and mineral admixtures can reduce cement consumption and promote the utilization of solid waste [16,23]. Nevertheless, most studies have examined RCP alone or binary systems containing RCP and a single mineral admixture. Systematic investigations of multicomponent systems simultaneously incorporating RCP, SF, GBFS, and FA, particularly their combined effects on engineering properties, wet carbonation behavior, and post-carbonation activity, remain limited.

When concrete is exposed to the atmosphere, CO_2_ diffuses into the system through pores and reacts with hydration products such as calcium hydroxide (Ca(OH)_2_) to form calcium carbonate (CaCO_3_), causing the pH of the pore solution to decrease [24]. Moderate carbonation can improve the system’s density by filling pores with CaCO_3_, thereby enhancing certain mechanical properties [25,26]; however, excessive carbonation leads to decalcification of the C–S–H gel and the formation of microcracks, which reduces the alkaline protective environment around the reinforcing bars and increases the risk of corrosion. At the same time, the carbonation process is essentially a form of CO_2_ mineralization and sequestration, enabling the long-term storage of CO_2_. Therefore, studying the carbonation behavior and carbon sequestration potential of cementitious materials from the perspective of CO_2_ sequestration is of great significance for the development of low-carbon building materials.

Currently, carbonation of recycled cementitious materials has attracted increasing attention as a promising route for both CO_2_ mineralization and construction-waste valorization. Previous studies have demonstrated that demolished concrete paste, recycled cement paste powder, recycled concrete fines, and concrete slurry waste contain calcium-bearing hydration products that can react with CO_2_ to form stable carbonate phases, thereby providing potential pathways for CO_2_ sequestration and supplementary cementitious material production [27,28,29,30]. In particular, wet carbonation has been shown to accelerate ion dissolution, promote CaCO_3_ precipitation, and modify the residual silica- or alumina-rich phases in recycled cementitious powders [29,31,32,33]. Meanwhile, carbonated recycled concrete fines and recycled cement paste powder have been increasingly investigated as emerging supplementary cementitious materials, and their effects on hydration kinetics, rheological behavior, mechanical properties, durability, and microstructural development have been systematically reviewed or experimentally evaluated [34,35,36,37]. In addition to recycled powder systems, accelerated carbonation and CO_2_ curing have also been widely used to improve recycled aggregate quality and to enhance the performance of cement-based materials containing RCP [38,39,40]. These studies have provided important insights into carbonation products, microstructural evolution, CO_2_ uptake capacity, and reuse potential of carbonated recycled materials. However, several research gaps remain. First, most previous studies have focused on recycled cement paste powder, recycled concrete fines, recycled aggregate carbonation, or binary cementitious systems, whereas the coupled effects of RCP, SF, GBFS, and FA in multicomponent recycled powder-based binders remain insufficiently understood. Because these constituents differ in chemical composition, reactivity, and dilution effects, their combined influence on hydration and phase development, and the consequent engineering performance, cannot be directly inferred from single-component or binary systems. In particular, the relationship between the hydration and phase characteristics of such multicomponent binders and their macroscopic mechanical response remains insufficiently clarified. Second, previous studies have generally examined carbonation products, CO_2_ uptake, or mechanical performance separately, while the relationships among alkaline-component leaching, pH evolution, carbonation kinetics, phase transformation, and CO_2_ sequestration capacity during wet carbonation have not been systematically clarified for such multicomponent binders. Third, although carbonated recycled powders have increasingly been considered for subsequent use in cement-based materials, limited attention has been paid to integrating post-carbonation activity with engineering performance and CO_2_ sequestration in a unified mixture-optimization framework. Therefore, a systematic investigation linking mixture composition, engineering performance, wet-carbonation behavior, CO_2_ sequestration potential, and post-carbonation reuse performance is still needed.

To address these gaps, this study developed a low-carbon recycled powder-based binder (LCRPB) incorporating RCP, SF, GBFS, and FA and systematically investigated its engineering performance, wet-carbonation behavior, CO_2_ sequestration potential, and post-carbonation activity. Specifically, a five-factor, five-level orthogonal experimental design was employed to evaluate the effects of the water-to-binder ratio and the contents of SF, GBFS, FA, and RCP on workability, mechanical properties, and hydration behavior. Wet-carbonation tests were subsequently conducted under controlled conditions to characterize alkaline-component leaching, pH evolution, carbonation kinetics, phase transformation, and CO_2_ sequestration capacity. The residual activity of the carbonated powder was further evaluated using the activity index as a preliminary indicator of its reuse potential in cement-based systems. Finally, a multi-objective matrix analysis was employed to identify a balanced formulation by jointly considering engineering performance, carbon-sequestration performance, and post-carbonation recyclability. The overall aim of this study is to clarify how the composition of multicomponent RCP-based binders governs their engineering and wet-carbonation behavior and to provide a quantitative basis for their low-carbon utilization and subsequent reuse. Based on these objectives, three hypotheses were proposed: (H1) mixture composition affects the engineering and hydration behavior of LCRPB; (H2) mixture composition influences wet-carbonation behavior and CO_2_ sequestration capacity; and (H3) carbonated LCRPB retains residual activity for subsequent reuse.

## 2. Test Protocol and Methods

### 2.1. Materials

**Figure 1:**
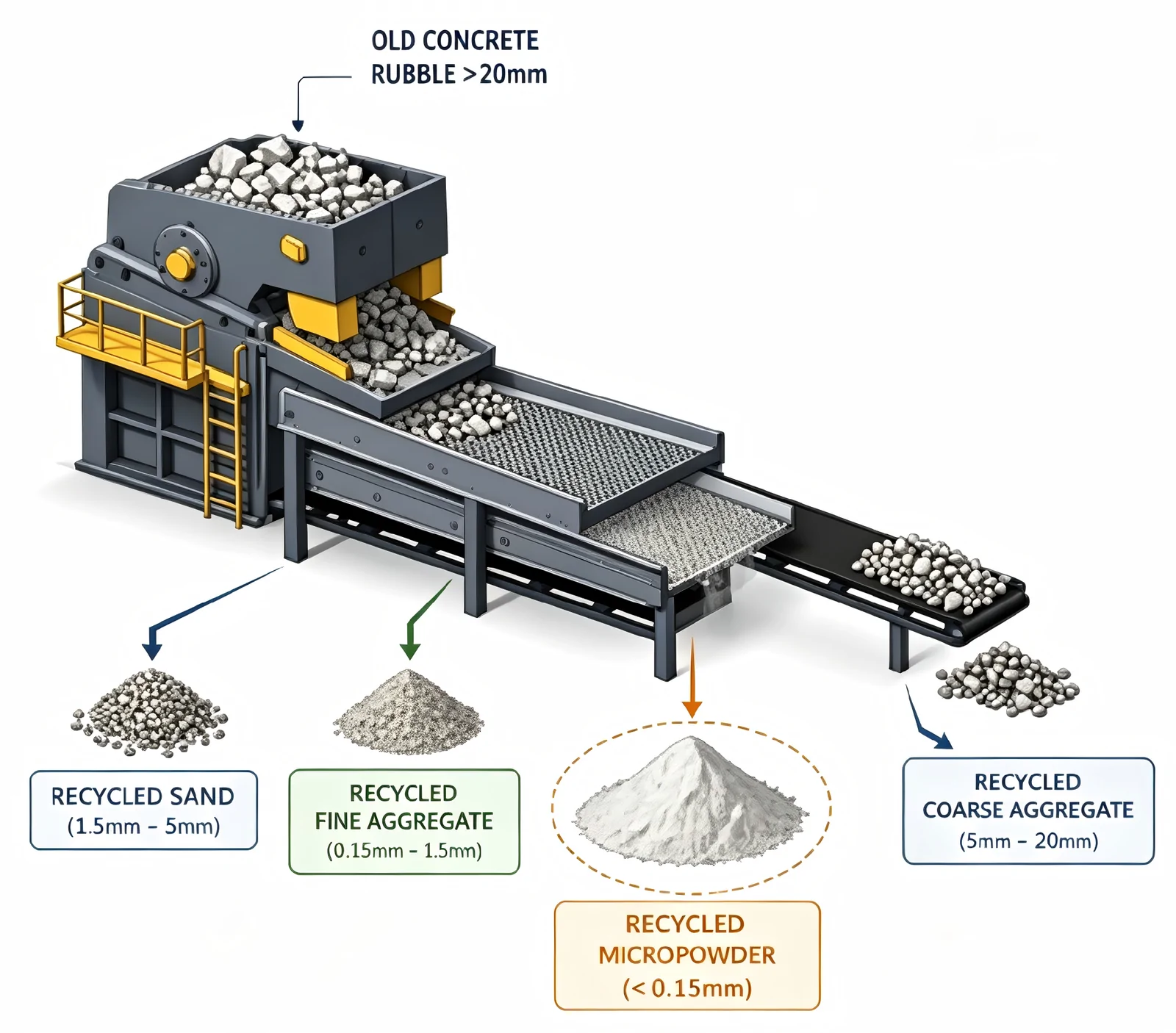
The RCP production process.

The cementitious materials used in this study include PO42.5 ordinary Portland cement (OPC), GBFS, FA, SF, and RCP obtained from waste concrete; Figure 1 illustrates the preparation process of RCP. Tap water was used in the experiments.

**Figure 2:**
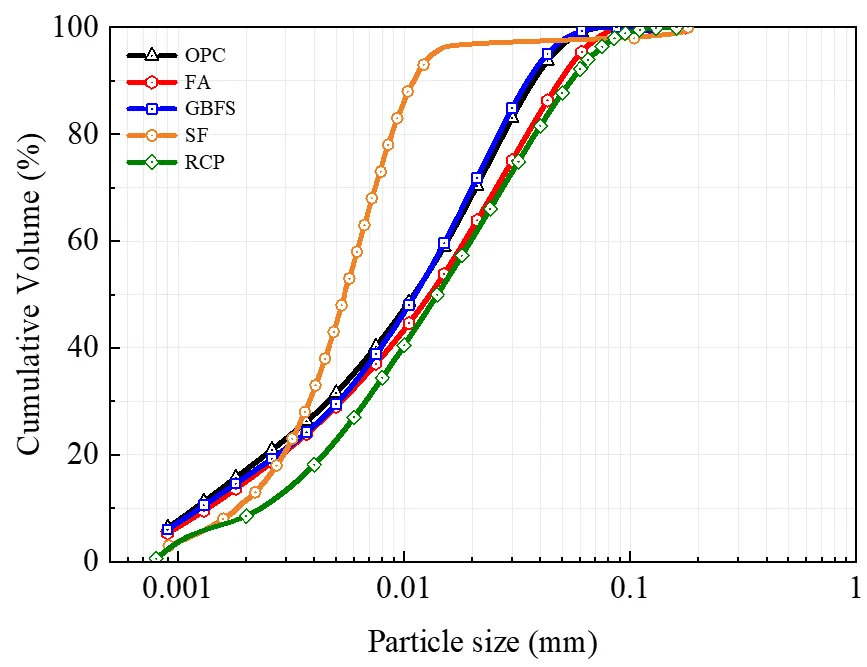
Particle size distribution of the cementitious material.

The chemical compositions of each material are shown in Table 1. As can be seen from the table, there are significant differences in the main chemical compositions of the cementitious materials. As a traditional cementitious material, OPC has the highest CaO content, reaching 63.21%, which determines the main characteristics of its hydration reaction. FA has a high SiO_2_ content of 45.05%, demonstrating strong pozzolanic activity. Additionally, its Al_2_O_3_ and Fe_2_O_3_ contents are 21.97% and 7.76%, respectively, indicating a chemical composition rich in aluminosilicate minerals, which may contribute to enhancing the system’s late-age strength. GBFS exhibits a relatively balanced SiO_2_ and CaO content of 32.34% and 39.69%, respectively, endowing it with both latent hydraulic activity and potential hydraulic properties. SF has an extremely high SiO_2_ content of 97.02%, significantly higher than that of other materials, reflecting its excellent pozzolanic activity. However, its CaO content is nearly zero at only 0.28%, suggesting that SF’s primary role may be to enhance the material’s density and reactivity. The chemical composition of RCP lies between that of FA and GBFS, with CaO and SiO_2_ contents of 29.02% and 33.55%, respectively. This suggests that RCP possesses some cementitious activity; additionally, its relatively high MgO content (3.83%) may play a role in improving the microstructure of concrete and enhancing durability. The particle size distribution of the cementitious materials is shown in Figure 2. SF is the finest-grained material among the cementitious materials, with a median particle size (D50) of 5.462 μm, enabling it to serve as an effective filler within the cementitious matrix.

### 2.2. Orthogonal Design of Experiments

In the LCRPB system, different components exert varying influences on the workability, mechanical properties, hydration reactions, and carbonation behavior of the paste. Among these, the water-to-binder ratio directly affects the paste’s flowability and hydration environment, serving as a critical fundamental parameter that determines the performance of the cementitious system; SF, GBFS, and FA, as common mineral admixtures, play distinct roles in early-stage reactivity, mid-to-late-stage strength development, and durability improvement; RCP, as a solid waste powder derived from waste concrete, not only affects the overall performance of the composite cementitious material through variations in its dosage but may also influence the system’s CO_2_ absorption potential. Therefore, this study selected the water-to-binder ratio (A), SF content (B), GBFS content (C), FA content (D), and RCP content (E) as orthogonal experimental factors, with five levels set for each factor. The ranges of these factors were selected by considering the different reactivities and functional characteristics of the constituents, as well as the need to cover sufficiently broad dosage intervals for evaluating their main effects. SF was limited to 0–8% because its high specific surface area and high pozzolanic reactivity allow it to exert pronounced effects at relatively low dosages, while excessive incorporation may substantially increase water demand and adversely affect workability. GBFS and FA were varied over 0–30% and 0–20%, respectively, to investigate their latent hydraulic and pozzolanic contributions over moderate replacement ranges. A wider range of 0–40% was adopted for RCP to evaluate both the potential beneficial effects at relatively low dosages and the dilution effects that may become dominant at higher replacement levels. Accordingly, the water-to-binder ratio ranged from 0.40 to 0.60, the SF content from 0% to 8%, the GBFS content from 0% to 30%, the FA content from 0% to 20%, and the RCP content from 0% to 40%; the specific levels for each factor are shown in Table 2. A five-factor, five-level orthogonal experimental design was adopted, and the mix designs were formulated based on the standard orthogonal table L25(5^6^), yielding a total of 25 test mix designs. The specific mix proportions for each test group are shown in Table 3. By conducting tests on engineering performance and accelerated carbonation for different mix designs, the effects of each factor on the carbon sequestration potential and formation mechanisms of the low-carbon composite cementitious materials were systematically analyzed.

### 2.3. Accelerated Carbonation Test Protocol

To quantify the CO_2_ sequestration potential of LCRPB, this study employed wet accelerated carbonation tests as an evaluation method to analyze the material’s reaction characteristics under controlled carbonation conditions. In the carbonation tests, the liquid-to-solid ratio and CO_2_ gas flow rate were selected as the primary control parameters. To establish a stable and reproducible carbonation evaluation system, RCP was chosen as the representative material for carbonation parameter screening tests. This is because RCP contains a certain amount of alkaline mineral phases and active calcium components, making it highly sensitive to CO_2_ carbonation reactions. Additionally, its relatively simple composition minimizes interference from multi-component composite systems on the carbonation process. A series of carbonation tests were conducted by varying the liquid-to-solid ratio and CO_2_ gas flow rate; the levels of each variable are shown in Table 4. During the screening of carbonation conditions, the pH of the solution was continuously monitored over time, and the CaCO_3_ content in the carbonation products was determined using thermogravimetric analysis (TGA) to evaluate the carbonation reaction efficiency and CO_2_ sequestration capacity of the material under different test conditions. Based on a comprehensive analysis of indicators such as carbonation rate, pH change characteristics, and CO_2_ absorption capacity, the optimal reaction conditions for the wet carbonation test were determined.

After determining the carbonation conditions, wet carbonation tests were conducted on the 25 sets of LCRPB samples obtained from the orthogonal experiments. During the carbonation process, the pH change curve of the solution was recorded, and key parameters of the carbonation reaction were extracted, including the pre-leaching stabilization time, the stable pH value during the pre-leaching stage, the pH decline rate during the carbonation stage, and the time required for carbonation to reach pH = 7. These parameters were used to characterize the leaching behavior of alkaline components, the kinetic characteristics of the carbonation reaction, and the extent of the carbonation reaction, respectively, thereby providing a basis for the mechanistic analysis of carbon sequestration potential.

Combining the mineral composition of the carbonation products and the amount of CO_2_ absorbed, the carbon sequestration potential of low-carbon composite cementitious materials is quantitatively evaluated. On this basis, to assess the reusability of the post-carbonation materials, the activity index of the carbonated powder is tested to analyze its potential for reuse in cement-based systems, thereby providing a reference for the application of recycled materials in low-carbon cementitious systems.

### 2.4. Test Method

#### 2.4.1. Flowability Test

The initial slump test procedure complies with standard GB/T 2419-2005 [41]. The slump of the fresh mortar is determined using a miniature slump cone (60 mm high, with a top diameter of 70 mm and a bottom diameter of 100 mm) on a 250 mm slump pan. The cone is first filled with fresh mortar, then lifted vertically, spreading the mortar over the top. The disk is tapped against the flow meter 25 times at a constant frequency within 25 s. For each formulation, the flowability test was independently repeated three times. In each test, two perpendicular spreading diameters were measured and averaged. The reported flowability was calculated as the mean of the three replicate tests, with the flow value recorded to the nearest 1 mm.

#### 2.4.2. Mechanical Property Testing

Mechanical property testing was conducted in accordance with the national standard GB/T 17671 [42]. The slurry prism specimens (40 mm × 40 mm × 160 mm) were cast in two layers, with each layer compacted using a vibrating table for 60 cycles. After casting, the specimens were placed in a standard curing chamber (T = 20 ± 2 °C, RH 95%) and demolded after 24 h. They were then kept in the standard curing chamber. Compressive and flexural strength tests were conducted at curing ages of 7 and 28 days. For each formulation and curing age, three 40 mm × 40 mm × 160 mm prism specimens were used for flexural strength testing, and the reported flexural strength was calculated as the mean of three measurements. After the flexural test, the six resulting half-prisms were subjected to compressive strength testing, and the reported compressive strength was calculated as the mean of six measurements. The error bars shown for the compressive and flexural strength results represent the standard deviations (SD) of the corresponding replicate measurements.

#### 2.4.3. Accelerated Carbonation

(1) Accelerated Carbonation Test

**Figure 3:**
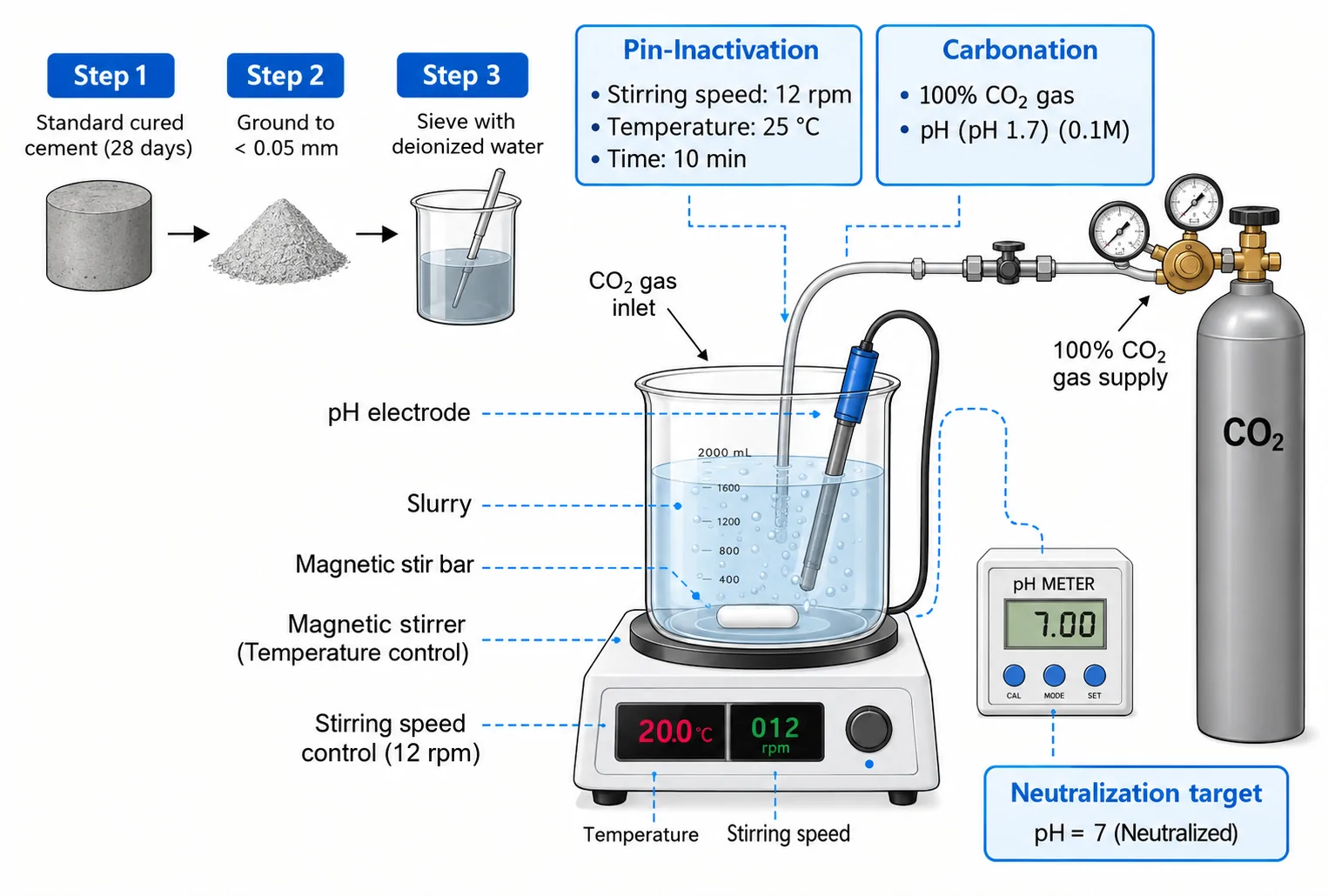
Schematic diagram of accelerated carbonation.

To characterize the CO_2_ sequestration potential and carbonation reaction characteristics of LCRPB under controlled conditions, this study employed a wet accelerated carbonation method for experimental investigation. A schematic diagram of the apparatus is shown in Figure 3. First, LCRPB slurry specimens that had been standard-cured for 28 days were crushed and ground to a particle size of less than 0.075 mm to prepare powdered samples for the carbonation test. This was done to increase the contact area between the material and CO_2_, thereby promoting a thorough carbonation reaction. Subsequently, the powdered samples were placed in a container and mixed with deionized water for pre-leaching treatment.

During the pre-leaching stage, a magnetic stirrer was used to maintain a stirring speed of 12 rpm and a temperature of 20 °C for 10 min to promote the leaching of alkaline components and establish a stable initial state. After pre-leaching, 100% CO_2_ was continuously introduced until the suspension pH decreased to 7, which was defined as the operational endpoint of the accelerated wet-carbonation test. This endpoint provided a reproducible basis for comparison among different formulations and indicated substantial neutralization of the dissolved alkalinity, rather than complete mineral carbonation. The occurrence and extent of mineral carbonation were further assessed by TGA/DTG and semi-quantitative XRD analyses. During wet carbonation, the complete pH–time evolution of each formulation was continuously monitored using a pH meter. The pre-leaching stabilization time, stable pH, pH decline rate, and time required to reach pH = 7 were determined directly from the corresponding pH–time curve and used for subsequent analysis.

(2) Calculation of the carbon sequestration capacity of LCRPB

X-ray diffraction (XRD) analysis was performed on the 25 orthogonal-test samples before and after wet carbonation. The mass of CaCO_3_ generated per unit mass of the 28-day hydrated powder during carbonation was calculated using Equation (1). Subsequently, based on the stoichiometric relationship between CaCO_3_ formation and CO_2_ uptake, the CO_2_ sequestration capacity per unit mass of the 28-day hydrated powder was calculated using Equation (2). Three parallel XRD measurements were conducted for each formulation. The CO_2_ sequestration capacity was calculated separately for each replicate using Equations (1) and (2), and the results are reported as the mean ± standard deviation (SD). The SD of the three replicate CO_2_ uptake values was used to characterize the experimental uncertainty associated with the semi-quantitative XRD-based estimation. Because the conversion from CaCO_3_ content to CO_2_ uptake is based on a fixed stoichiometric relationship, the variability in the semi-quantitative XRD results was directly propagated to the calculated CO_2_ sequestration capacity.

(1) $$m_{CaCO_{3}}=m_{0}\times \left(\omega_{CaCO_{3}}/100\right)$$

(2) $$m_{CO_{2}}=\frac{m_{CaCO_{3}}\times 44}{100\times m_{0}}$$

In the equation: $m_{{CaCO}_{3}}$—Mass of CaCO_3_ produced by the carbonation reaction, g; $m_{0}$—represents the mass of the 28-day hydrated powder used for the carbonation test; in this study, $m_{0}$ was 10 g; $\omega_{{CaCO}_{3}}$—The change in the mass fraction of CaCO_3_ before and after carbonization; $m_{{CO}_{2}}$—the amount of CO_2_ sequestered per unit mass of the 28-day hydrated powder, g. Accordingly, the CO_2_ sequestration capacity reported in this study is normalized to the mass of the 28-day hydrated powder and is expressed as g CO_2_/100 g hydrated powder.

It should be noted that the CO_2_ sequestration capacity calculated by Equations (1) and (2) represents an XRD-based estimate derived from the increase in crystalline CaCO_3_ retained in the analyzed solid fraction. During wet carbonation, other Ca-bearing phases may also participate in carbonation, while part of the carbonate species may remain dissolved in the liquid phase or precipitate outside the recovered solid fraction. In addition, semi-quantitative XRD primarily reflects crystalline carbonate phases and may not fully account for poorly crystalline or amorphous carbonation products. Therefore, the calculated CO_2_ uptake should be interpreted as an estimate of the solid-phase crystalline carbonate sequestration under the present experimental conditions rather than as a complete CO_2_ mass balance of the gas–liquid–solid system.

#### 2.4.4. Recycling Test

To evaluate the reuse potential of LCRPB powder after wet carbonation, the activity index (AI) was employed as a preliminary strength-based indicator of its residual cementitious activity in cement-based systems. The test was conducted in accordance with the Test Method for Strength of Cement Mortar. Following wet carbonation at a liquid-to-solid ratio of 5, a CO_2_ flow rate of 3 L/min, and a temperature of 20 °C until the suspension pH reached 7, the carbonated powder was dried, ground, and sieved through a 0.075 mm sieve before reuse. In the test mortar, the carbonated powder was used to replace 30% of the reference cement by mass, and the water-to-binder ratio was maintained at 0.50. The reference mortar consisted of 450 g of reference cement, 1350 g of ISO standard sand, and 225 g of water. For both the reference mortar and the mortar containing carbonated powder, three 40 mm × 40 mm × 160 mm prism specimens were prepared. After casting, the specimens were kept for 24 h before demolding and were subsequently cured for 28 days under standard curing conditions at a temperature of (20 ± 1) °C and a relative humidity of no less than 90%. After the curing period, the three prism specimens in each group were subjected to flexural splitting, producing six half-prism specimens for compressive strength testing. The compressive strength test was performed using a compression testing machine at a loading rate of 2400 ± 200 N/s. The reported compressive strength for each group was calculated as the mean of six measurements (*n* = 6), and the corresponding mean compressive strengths of the test mortar and reference mortar were used to calculate the activity index according to Equation (3):

(3) $$AI=\frac{f_{c,test}}{f_{c,benchmark}}\times 100\%$$

In the equation: AI—Activity Index, %; $f_{c,test}$—28-day compressive strength of mortar mixed with carbonized powder, MPa; $f_{c,benchmark}$—28-day compressive strength of the reference mortar, in MPa.

#### 2.4.5. Microscopic Testing

(1) XRD Testing

XRD was used to characterize the 25 orthogonal-test samples before and after wet carbonation. For each formulation, three replicate XRD measurements were performed before and after carbonation (n = 3 for each state). The measurements were conducted using Cu-Kα radiation at 40 kV and 15 mA (Rigaku Corporation, Tokyo, Japan). Prior to analysis, the samples were ground to a D50 of approximately 20 μm and dried to constant mass to ensure consistency of the measurements. The scanning range was 2θ = 5–90°, with a step size of 0.05° and a scanning rate of 0.05°/s. The diffraction patterns were analyzed using JADE 6.0 (Materials Data Inc., Livermore, CA, USA) for phase identification and semi-quantitative estimation of the crystalline phase contents. One representative diffraction pattern from the three replicate measurements was selected for presentation, whereas all three replicate measurements were used for the semi-quantitative estimation of CaCO_3_ content. The mean value obtained from the three measurements was subsequently used to estimate the CO_2_ sequestration capacity, as described in Section 2.4.3.

(2) Isothermal Calorimetry

The hydration heat of the LCRPB formulations was measured using a TAM Air isothermal calorimeter (TA Instruments, New Castle, DE, USA). The cementitious constituents were proportioned according to the designed formulations and mixed with water at the corresponding water-to-binder ratio. Immediately after mixing, the fresh paste was transferred into the calorimeter sample cell and placed in the calorimeter maintained at 25 °C. The heat-flow rate and cumulative heat release were continuously recorded for 72 h. The resulting heat-flow and cumulative-heat curves were used to characterize the hydration kinetics of the different LCRPB formulations, including the evolution of the main exothermic stages and the overall heat release during the monitoring period.

(3) TGA Testing

TGA was performed using a Netzsch STA 449 F3 thermogravimetric analyzer (NETZSCH-Gerätebau GmbH, Selb, Germany) to characterize the carbonation products of RCP under the different wet-carbonation conditions described in Section 2.3. Approximately 10 mg of each sample was placed in an alumina crucible and heated from room temperature to 1000 °C at a constant heating rate of 10 °C/min under an air atmosphere. Both TG and derivative DTG curves were used for analysis. The mass-loss region of approximately 30–250 °C was associated mainly with the release of bound water and decomposition of gel-related products, whereas the mass loss in the range of approximately 700–850 °C was associated with the decomposition of CaCO_3_. The carbonate-related mass loss in this latter temperature interval was used to compare the extent of carbonate formation under different liquid-to-solid ratios and CO_2_ flow rates.

### 2.5. Carbon Footprint Assessment

To quantitatively evaluate the low-carbon performance of LCRPB, a screening-level carbon footprint assessment was conducted using 1 kg of dry binder as the functional unit, with a 100% ordinary Portland cement (OPC) binder as the reference. The assessment boundary considered the material-related carbon emissions associated with the production or processing of OPC, SF, GBFS, FA, and RCP. The carbon emission factors and their corresponding data sources are summarized in Table 5.

The material-related carbon footprint of the binder was calculated using Equation (4):

(4) $$C_{mat}=\sum_{i=1}^{n}x_{i}EF_{i}$$

where $C_{mat}$ is the embodied carbon of the binder, kg CO_2_-eq/kg binder; $x_{i}$ is the mass fraction of constituent i in the binder; and ${EF}_{i}$ is the corresponding emission factor, kg CO_2_-eq/kg material.

The material-related carbon reduction relative to the 100% OPC reference was calculated using Equation (5):

(5) $$R_{mat}=\frac{C_{OPC}-C_{mat}}{C_{OPC}}\times 100\%$$

where $C_{OPC}$ is the carbon footprint of the 100% OPC reference binder, kg CO_2_-eq/kg binder, and $R_{mat}$ is the material-related carbon reduction ratio, %.

For a selected factor-level combination that was not directly included as an individual mixture in the orthogonal array, its CO_2_ sequestration capacity was estimated using the additive main-effect model as Equation (6):

(6) $$m_{co_{2},pred}=\bar{K}+\sum_{i=1}^{n}\left(K_{i}-\bar{K}\right)$$

where $m_{{CO}_{2},pred}$ is the predicted CO_2_ sequestration capacity of the selected combination, $\bar{K}$ is the overall mean CO_2_ sequestration response of the orthogonal experiment, and $K_{i}$ is the mean response corresponding to the selected level of factor i.

The present assessment represents a screening-level material-related carbon footprint analysis rather than a complete life-cycle assessment. The assessment boundary includes the production or processing of the constituent materials but does not include transportation, mixing, curing, or the energy consumption associated with the laboratory-scale wet-carbonation process. In addition, because the experimentally determined CO_2_ sequestration capacity was normalized to the mass of the 28-day hydrated powder, whereas the carbon-footprint assessment was based on the initial dry-binder mass, the two indicators were evaluated separately and were not directly combined into a net carbon-footprint calculation.

### 2.6. Statistical Analysis Methods

Range analysis and analysis of variance (ANOVA) were used to evaluate the effects of water-to-binder ratio, silica fume, granulated blast furnace slag, fly ash, and RCP admixture levels on various performance indicators. Range analysis was based on the mean values of the test indicators at different levels of each factor; the range is defined as the difference between the maximum and minimum mean values of a given factor across its various levels, and is used to compare the relative magnitudes of the main effects of each factor. Building on this, ANOVA was used to further evaluate the impact of each factor on the variability of the test results. The sum of squares (SS) represents the variability in the test results caused by the corresponding factor; the degrees of freedom (DF) are determined by the number of levels for each factor. In this study, each factor was set at five levels, so the degrees of freedom for each factor were 4; the mean square (MS) is the ratio of the sum of squares to the corresponding degrees of freedom; and the F-value is the ratio of the mean square for the factor to the mean square for the error.

Range analysis was used to compare the relative magnitude and ranking of the main effects of the investigated factors across different factor levels, whereas ANOVA was employed to assess the statistical significance of these effects. The two approaches were interpreted jointly, with range analysis providing a descriptive assessment of relative factor responses and ANOVA providing statistical inference regarding the observed differences. It should be noted that the L25 orthogonal design provides four residual degrees of freedom for the ANOVA error term after accounting for the five five-level factors, which limits the statistical power for detecting moderate effects. The ANOVA results were therefore interpreted in conjunction with the range analysis and the observed response trends. The adopted orthogonal design remains effective for screening the main effects of multiple factors with a limited experimental burden, although its ability to resolve factor interactions and experimental error is comparatively constrained.

### 2.7. Use of Generative AI for Figure Preparation

Generative AI software, ChatGPT (GPT-5, OpenAI), was used solely to assist in the preparation of the schematic illustrations presented in Figure 1, Figure 3 and Figure 15. The scientific content of these figures, including the experimental procedures, process sequence, apparatus configuration, labels, chemical species, and mechanistic interpretation, was determined by the authors based on the experimental design, experimental observations, and analysis conducted in this study. All AI-generated visual elements were subsequently reviewed and edited by the authors to ensure their scientific accuracy and consistency with the research. Generative AI was not used to generate, modify, or analyze any experimental data or quantitative results presented in this study.

## 3. Results and Discussion

### 3.1. Work Performance

**Figure 4:**
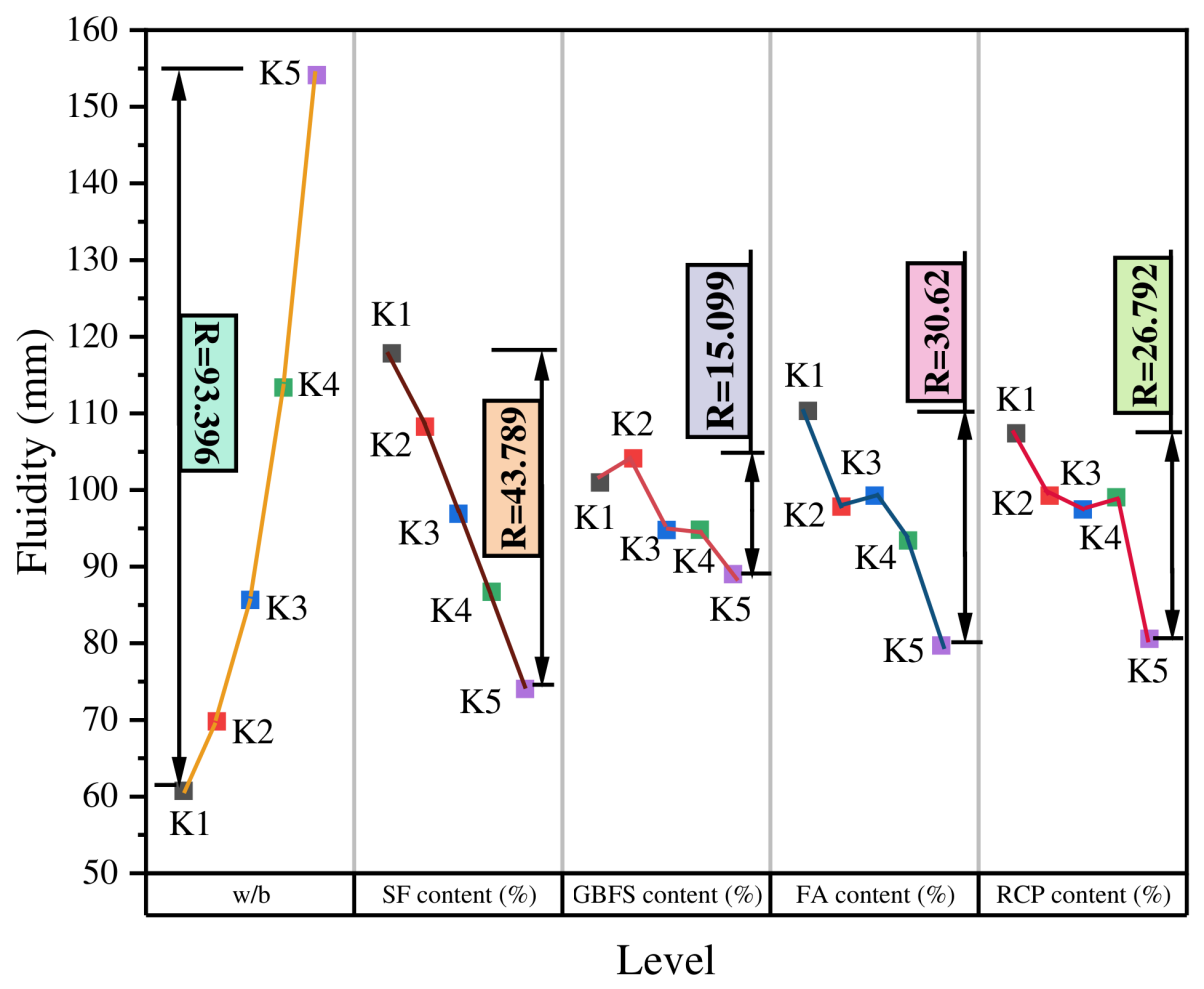
Effect of each factor in the orthogonal experiment on the flowability of LCRPB.

The effects of different factors on the slurry flowability of LCRPB are shown in Figure 4. As the water-to-binder ratio increased, slurry flowability gradually increased. In contrast, increasing SF content caused a nearly linear decrease in flowability due to its ultrafine particle size and high specific surface area, which increased the water demand of the system. The flowability showed slight fluctuations with increasing FA, GBFS, and RCP contents, but exhibited an overall decreasing trend. Previous studies reported that RCP possesses irregular particles, rough surfaces, and high water absorption, which negatively affects workability [19]. In this study, the flowability decreased by 7.57% and 24.95% when the RCP content increased to 10% and 40%, respectively. The range analysis results (Figure 4) indicate that the influence of each factor on flowability follows the order: W/B > SF content > FA content > RCP content > GBFS content (A > B > D > E > C). Since higher flowability is preferred, the optimal factor-level combination was determined as A5B1C2D1E1, corresponding to a water-to-binder ratio of 0.60, an SF content of 0%, a GBFS content of 7.5%, an FA content of 0%, and an RCP content of 0% (A5B1C2D1E1). ANOVA results (in Table 6) further showed that W/B and SF exerted statistically significant effects on slurry flowability, whereas the effects of GBFS, FA, and RCP did not reach statistical significance within the investigated factor levels.

### 3.2. Mechanical Properties

#### 3.2.1. Compressive Strength

**Figure 5:**
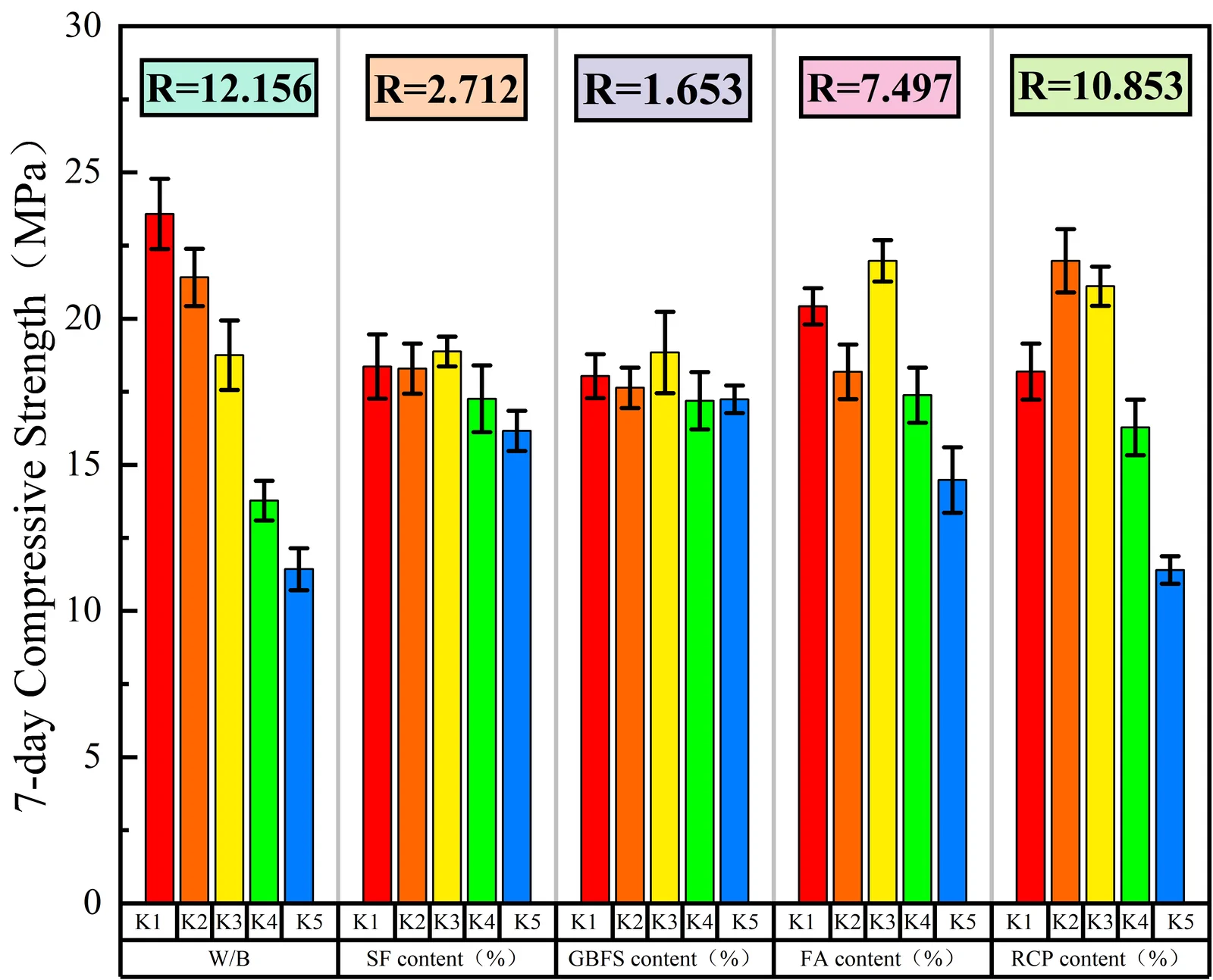
Compressive strength at 7 days.

**Figure 6:**
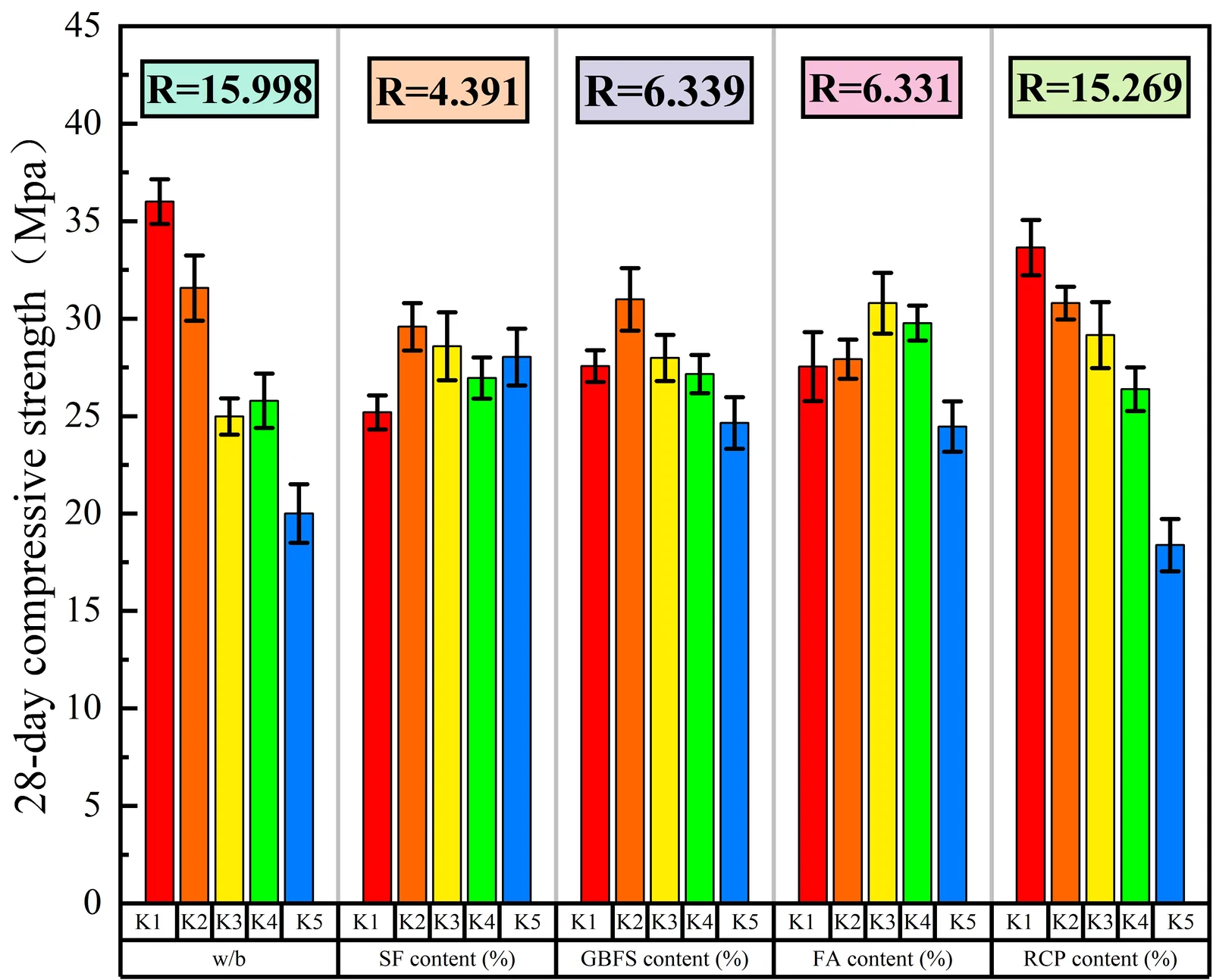
Compressive strength at 28 days.

Figure 5 and Figure 6 present the effects of different factors on the 7-day and 28-day compressive strengths of LCRPB paste. Overall, the 28-day compressive strength showed a marked decrease with increasing water-to-binder ratio, while only slight differences were observed between ratios of 0.50 and 0.55. This behavior is related to the combined effects of hydration degree and pore structure evolution. As shown in Figure 9, the hydration heat first increased and then decreased with increasing water-to-binder ratio. At low ratios, insufficient free water limited hydration, whereas an appropriate increase promoted hydration and enhanced matrix densification. However, excessive water diluted the slurry and increased pore volume, resulting in reduced structural compactness and compressive strength. With increasing RCP content, the 28-day compressive strength showed an overall decreasing trend, which was mainly attributed to the low reactivity and dilution effect of RCP. Figure 9 indicates that the hydration heat decreased almost linearly with increasing RCP content, suggesting that RCP contributed little to hydration while reducing the amount of effective cementitious material. When the RCP content increased from 30% to 40%, the largest decrease in strength was observed, reaching 30.36%.

In contrast, increasing SF, GBFS, and FA contents caused the 28-day compressive strength to first increase and then decrease. At low dosages, SF accelerated hydration and improved matrix densification through nucleation and pozzolanic effects. GBFS contributed to later-age hydration under alkaline conditions, while FA mainly enhanced long-term performance through pozzolanic reactions. However, excessive mineral admixture content weakened hydration due to dilution effects and limited reactivity, resulting in insufficient hydration products and reduced strength. The addition of 2% SF, 7.5% GBFS, and 10% FA increased the 28-day compressive strength by 17.43%, 12.43%, and 11.83%, respectively. Range analysis (Figure 6) showed that the relative influence of the investigated factors on 28-day compressive strength followed the order W/B > RCP content > GBFS content > FA content > SF content (A > E > C > D > B). ANOVA results (in Table 7) identified W/B as the factor exerting a statistically significant effect on 28-day compressive strength, whereas the remaining compositional factors exhibited different levels of response variation within the investigated factor range.

#### 3.2.2. Flexural Strength

**Figure 7:**
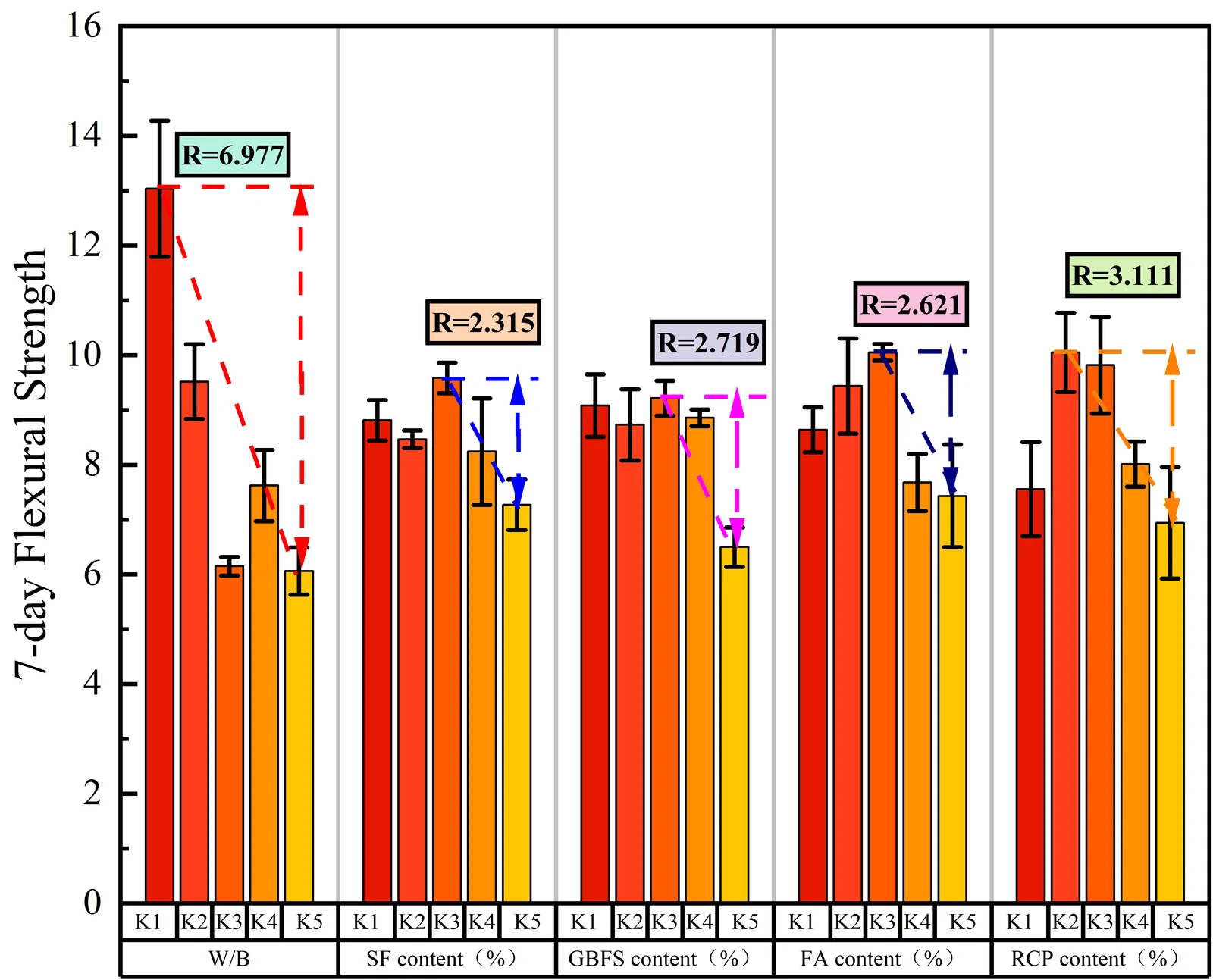
Effect of various factors in an orthogonal experiment on the 7-day flexural strength of LCRPB.

**Figure 8:**
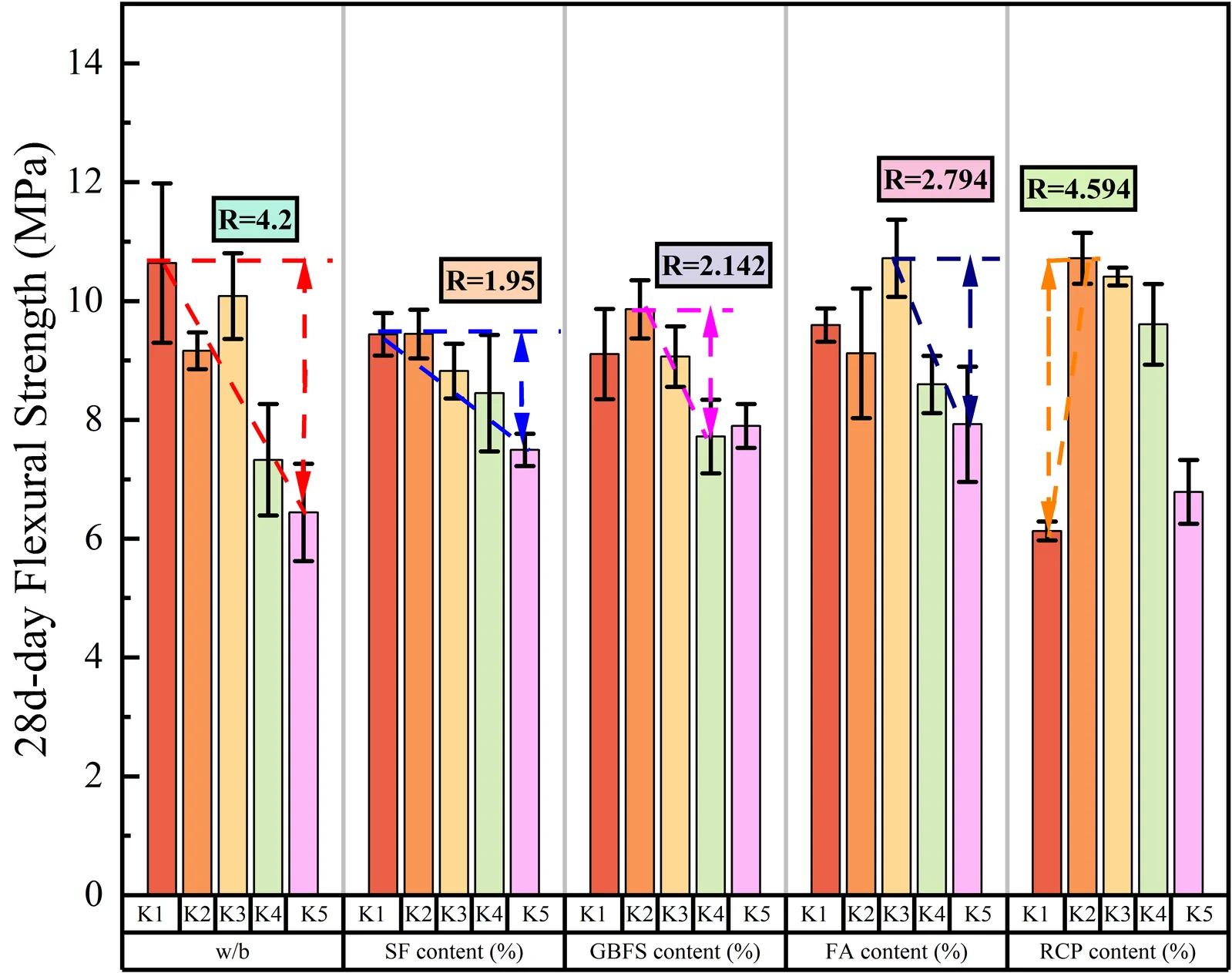
Effect of various factors in an orthogonal experiment on the 28-day flexural strength of LCRPB.

Figure 7 and Figure 8 present the effects of various factors on the 7-day and 28-day flexural strengths of LCRPB paste, respectively. As the contents of SF, GBFS, and FA increased, the flexural strength initially increased and then decreased. For the 7-day flexural strength, all three mineral admixtures reached their highest values at the third dosage level. For the 28-day flexural strength, SF and GBFS showed the highest values at the second dosage level, whereas FA reached its highest value at the third dosage level. This behavior was mainly attributed to the different reaction characteristics of the mineral admixtures. SF and GBFS possess relatively high pozzolanic or latent hydraulic activity, which promotes early-age strength development, whereas FA participates more extensively in later-age reactions, resulting in a more pronounced contribution to later-age strength. RCP content significantly affected the 28-day flexural strength. With increasing RCP content, the 28-day flexural strength initially increased and then decreased, reaching its highest value at an RCP content of 10%. This improvement may be attributed to the filling effect of fine RCP particles and the presence of residual unhydrated cementitious components. However, at higher RCP contents, the dilution effect and relatively low reactivity of RCP became more pronounced, leading to a reduction in flexural strength. Range analysis showed that the relative influence of the investigated factors on 28-day flexural strength followed the order RCP content > W/B > FA content > GBFS content > SF content (E > A > D > C > B). When 28-day flexural strength was taken as the optimization objective, the preferred factor-level combination was A1B2C2D3E2. ANOVA results (in Table 8) further identified statistically significant effects of RCP content, W/B, and GBFS content, while the effects associated with SF and FA did not reach statistical significance.

### 3.3. Heat of Hydration

**Figure 9:**
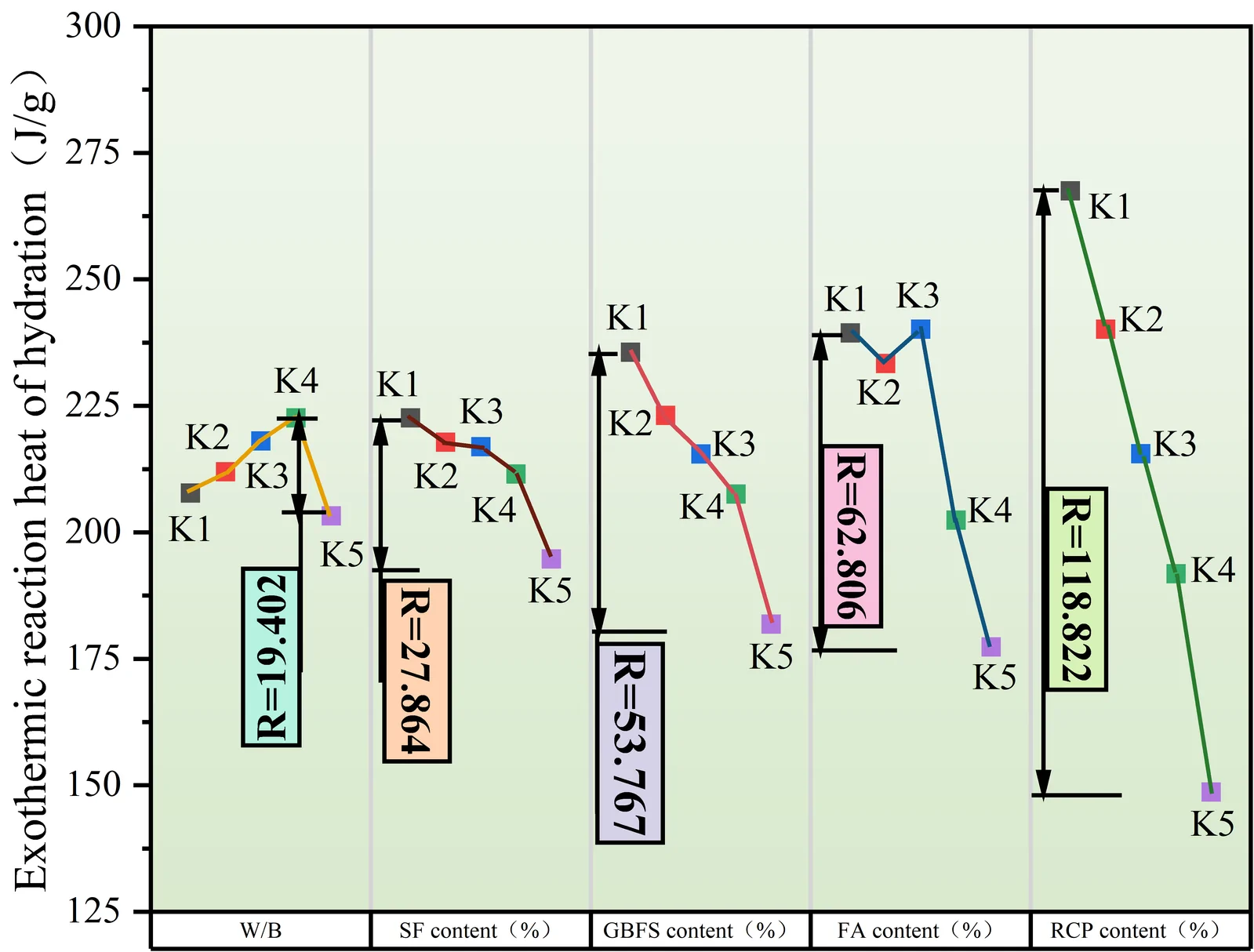

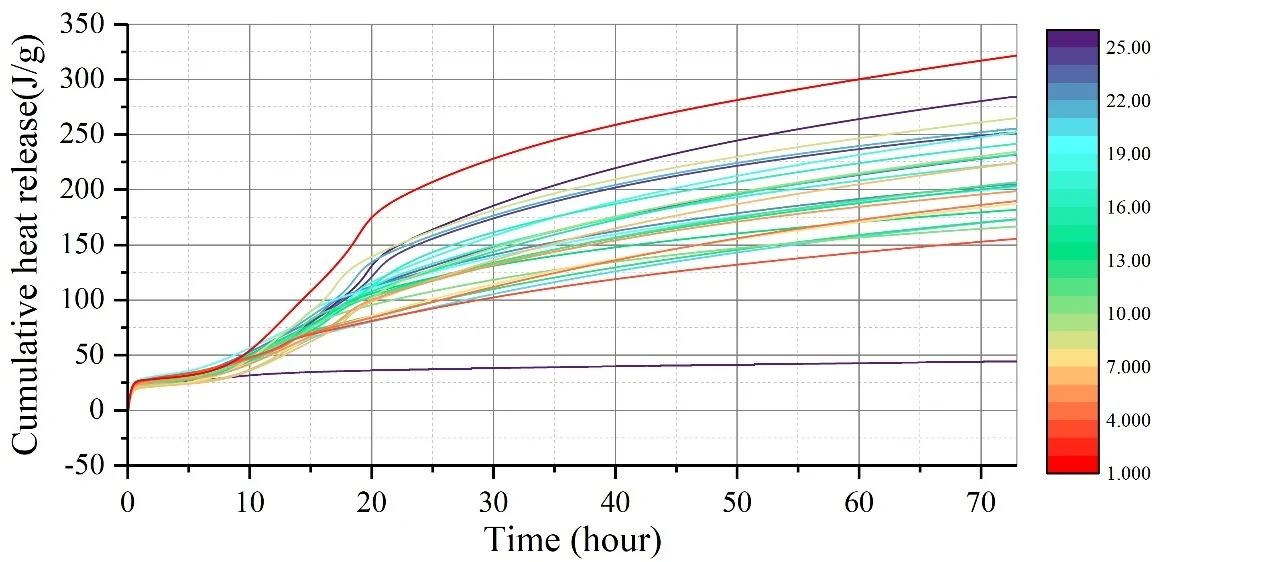

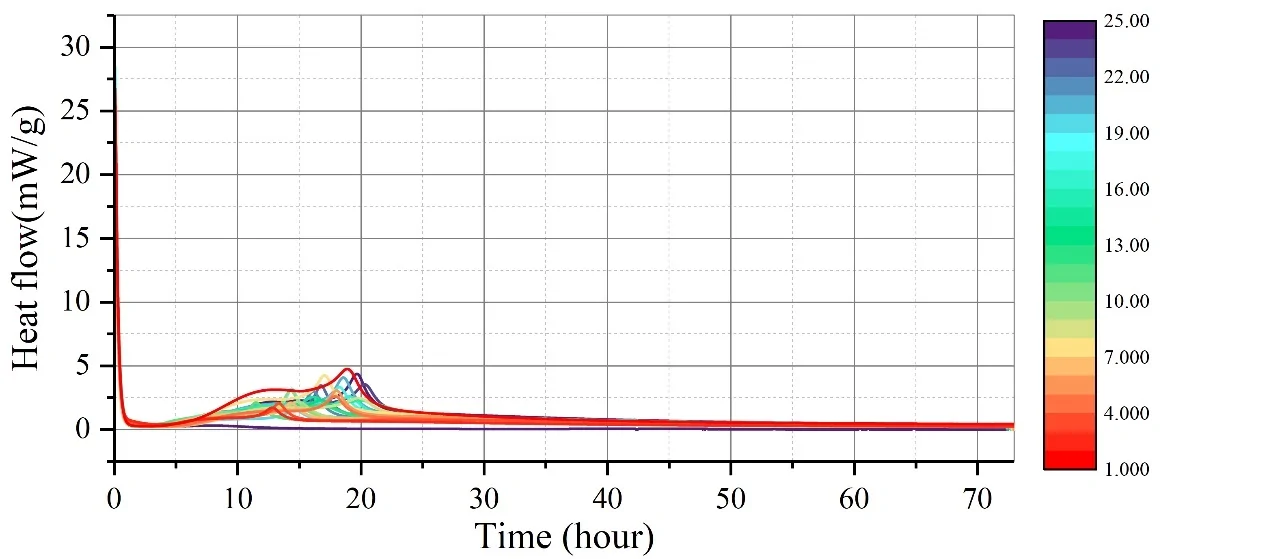
Effect of factors in an orthogonal experiment on the heat of hydration of LCRPB.

The characteristics of hydration heat constitute the intrinsic kinetic basis for the evolution of compressive and flexural strengths; this section is dedicated to elucidating the mechanisms underlying these changes in mechanical properties. According to the range analysis (Figure 9), the relative magnitude of the factor effects on the hydration heat of LCRPB followed the order RCP content > FA content > GBFS content > SF content > W/B (E > D > C > B > A), with RCP exhibiting the largest range-based effect within the investigated levels.

The hydration heat decreased approximately linearly with increasing RCP content. This behavior is mainly attributed to the relatively low reactivity of RCP and the replacement of highly reactive OPC, indicating that the dilution effect dominates over the possible filling and nucleation effects of RCP within the 72 h hydration period. This trend is consistent with the decrease in 28-day compressive strength observed with increasing RCP content. The effect of the water-to-binder ratio was non-monotonic: insufficient water at low W/B restricted hydration, whereas a moderate increase improved water availability and ion transport; at higher W/B, however, the additional water no longer produced a corresponding increase in hydration heat. SF, GBFS, and FA also showed different responses because of their distinct reaction characteristics. SF mainly contributes through its fine-particle and pozzolanic effects, GBFS through latent hydraulic reactions under alkaline conditions, whereas FA reacts relatively slowly during the early hydration period. At higher replacement levels, the reduction in OPC content gradually outweighs these beneficial effects, resulting in lower heat release. These hydration characteristics are consistent with the corresponding variations in compressive and flexural strengths.

### 3.4. Optimal Parameters for Wet Carbonation

The efficiency of wet carbonation is influenced not only by the composition of the reaction system, but also by the liquid-phase environment and CO_2_ mass transfer conditions. To avoid interference from multicomponent interactions in the analysis of carbonation mechanisms, RCP was first selected as a representative material to optimize the liquid-to-solid ratio and CO_2_ flow rate. The optimized conditions were subsequently applied to the carbonation study of the LCRPB system.

**Figure 10:**
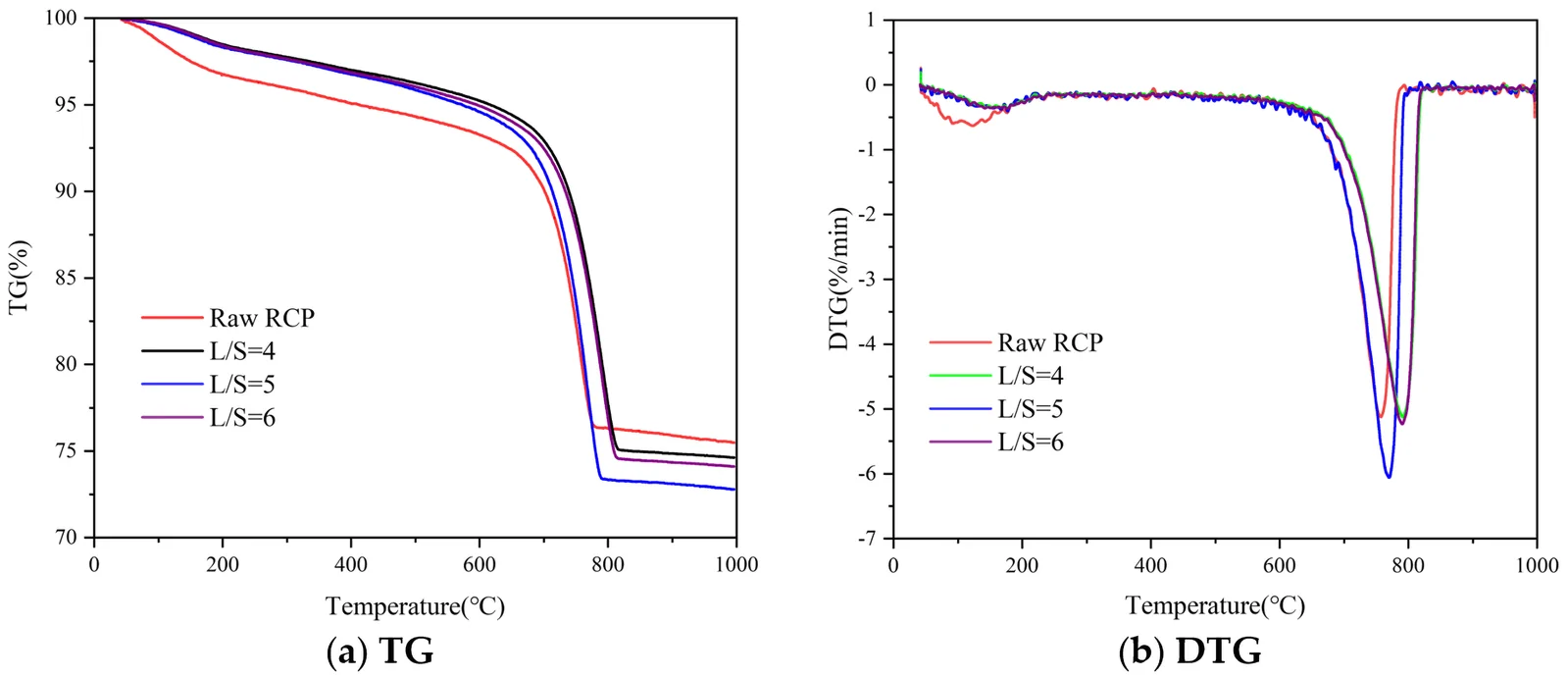
Thermogravimetric analysis of RCP after wet carbonation at different liquid-to-solid ratios.

**Figure 11:**
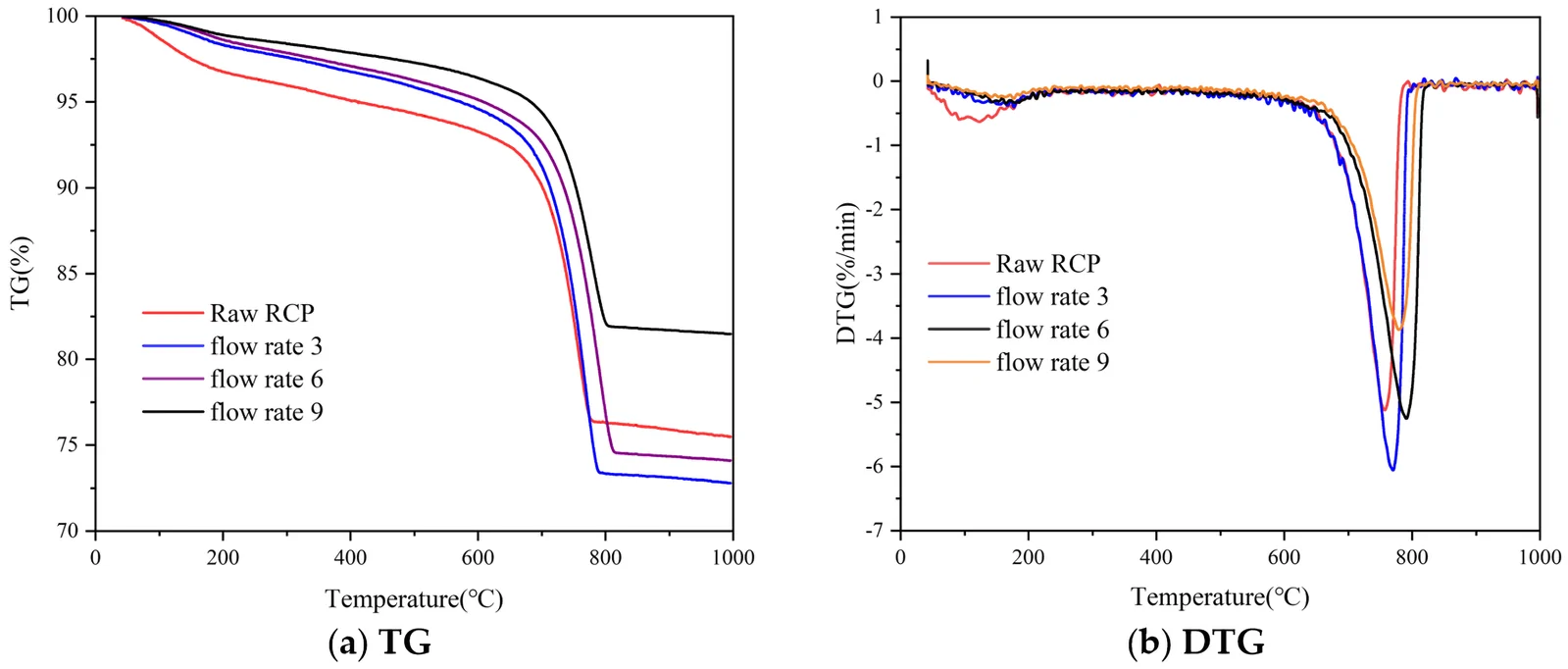
Thermogravimetric analysis of RCP after wet carbonation at different CO_2_ flow rates.

TGA results of RCP carbonated under different liquid-to-solid ratios and gas flow rates are shown in Figure 10 and Figure 11. To ensure comparability, the carbonation time was fixed at 20 min. Compared with the uncarbonated RCP, the carbonated samples exhibited greater mass loss in the carbonate-decomposition region, indicating increased CaCO_3_ formation during wet carbonation. As the liquid-to-solid ratio increased, the total weight loss first increased and then decreased. At low ratios, insufficient dispersion limited the carbonation reaction. At higher liquid-to-solid ratios, the increased liquid volume may have promoted the transfer of carbonate species into the liquid phase and/or the formation of carbonate precipitates that were not fully retained in the recovered solid fraction. Two major DTG peaks were observed: the first peak at 30–250 °C corresponded to the evaporation of bound water and decomposition of gel products, while the second peak at 700–850 °C was associated with CaCO_3_ decomposition. The significant reduction in the first peak after carbonation indicated substantial consumption of gel products during carbonation. The highest carbonate decomposition peak was obtained at a liquid-to-solid ratio of 5, suggesting the greatest carbonate formation under this condition. A similar trend was observed for different CO_2_ flow rates. Increasing the flow rate promoted carbonation initially; however, when the CO_2_ flow rate exceeded 3 L/min, the carbonate-related mass loss decreased, suggesting that part of the carbonate species may have remained in the liquid phase or formed precipitates outside the recovered solid fraction. The CaCO_3_ weight loss results (Table 9) demonstrate that the optimal carbonation condition was achieved at a liquid-to-solid ratio of 5 and a CO_2_ flow rate of 3 L/min.

It should be emphasized that the carbonation conditions adopted in the present study were intentionally accelerated to establish a controlled and reproducible framework for evaluating the relative carbonation response of the investigated materials. In particular, the use of 100% CO_2_ does not directly represent practical industrial carbonation environments, in which CO_2_ concentration, flue-gas composition, gas–liquid mass-transfer characteristics, residence time, moisture conditions, and reactor configuration may substantially influence carbonation kinetics and the attainable CO_2_ uptake. Accordingly, the carbonation performance obtained under the present conditions should be interpreted primarily as a comparative measure of carbonation potential rather than as a direct prediction of industrial-scale performance. Further validation under lower CO_2_ concentrations and, preferably, representative flue-gas conditions is therefore required to assess the transferability of the laboratory-scale findings to practical carbonation processes.

### 3.5. Changes in Hydration Products Before and After Carbonation

(1) XRD Analysis Before Carbonation

**Figure 12:**
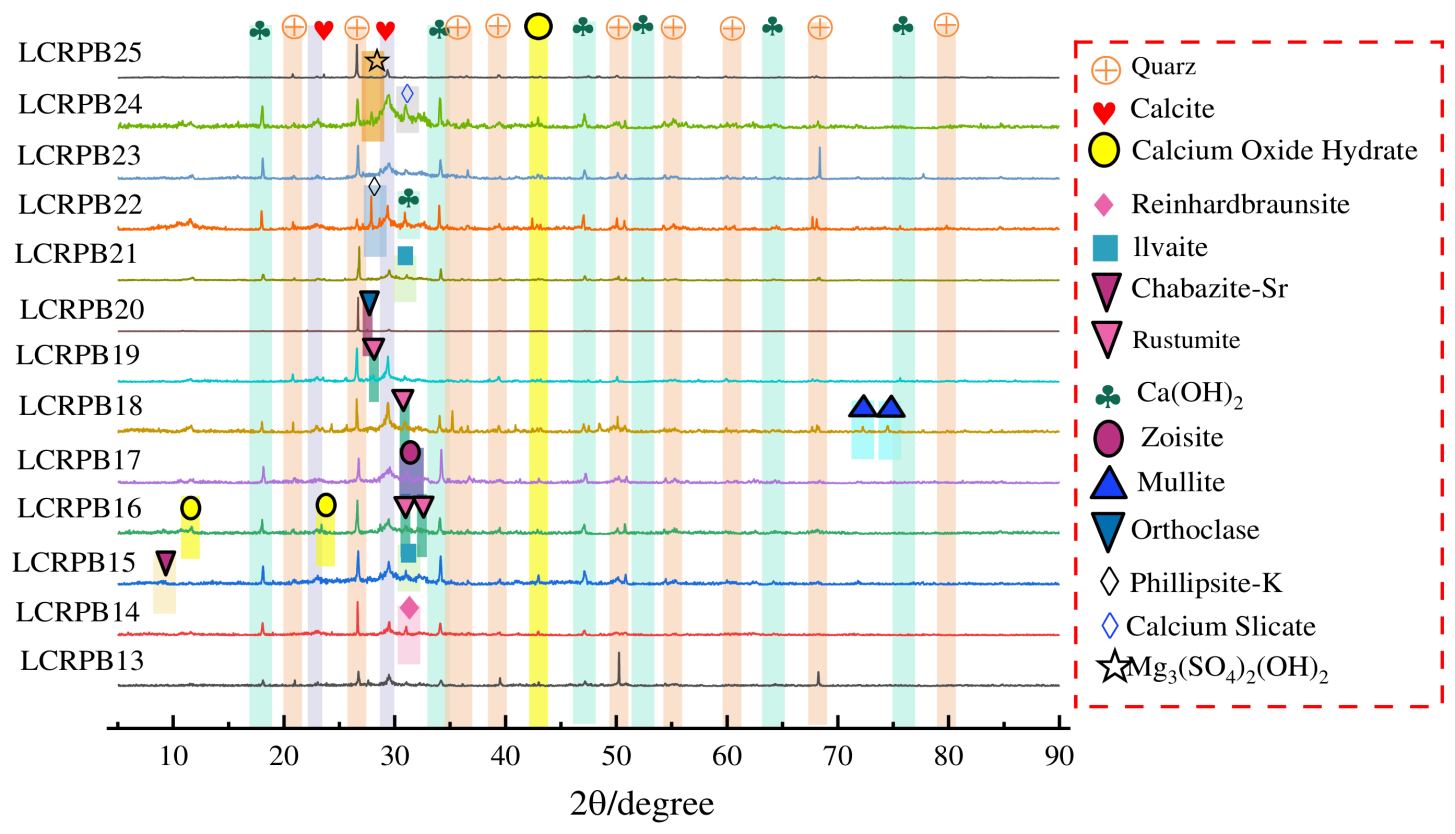

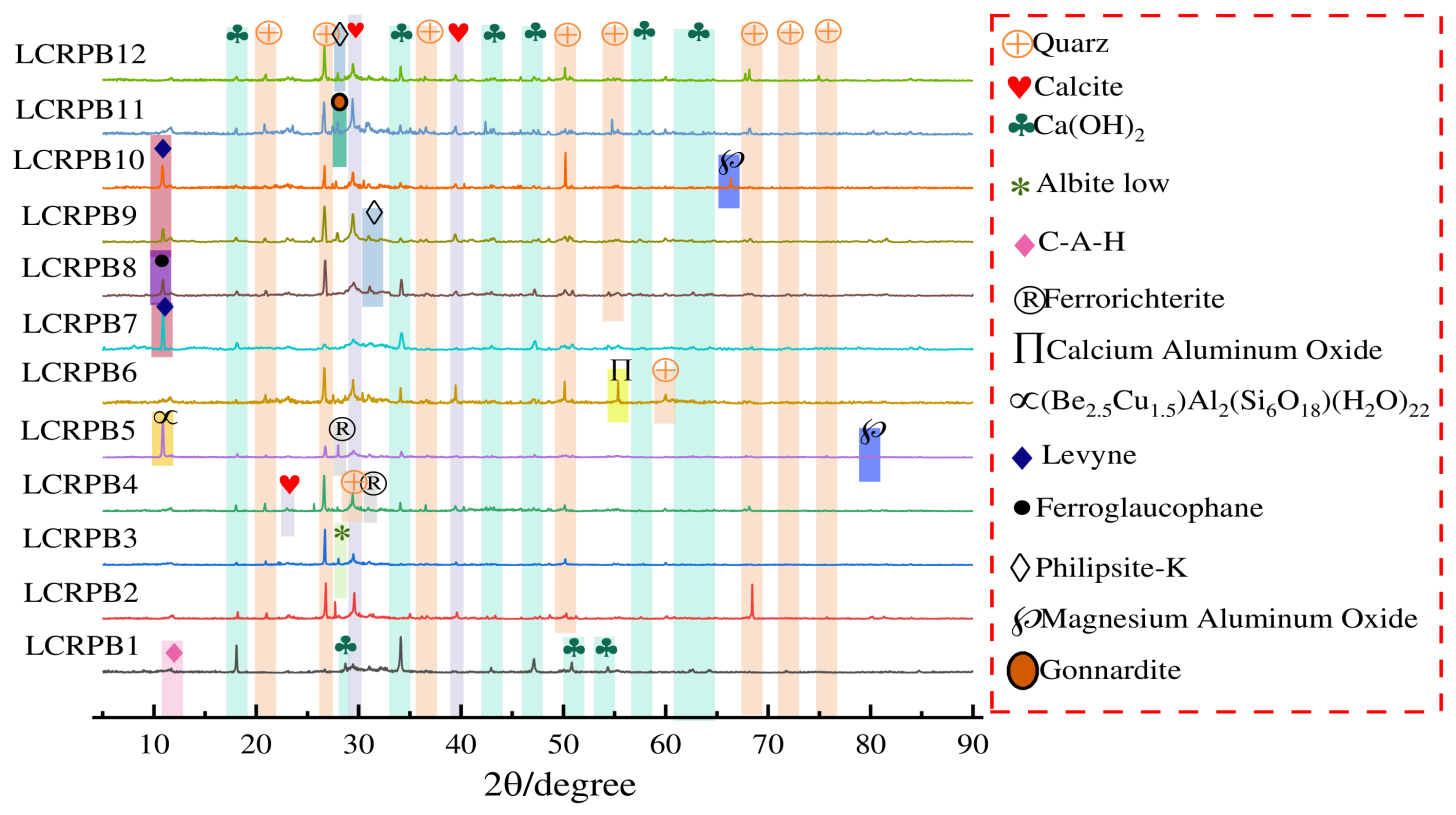
XRD spectrum of LCRPB after 28 days of hydration.

The XRD results for the samples in each group of the orthogonal experiments are shown in Figure 12. As can be seen from the figure, the main hydration products in the pure cement system are calcium hydroxide (Ca(OH)_2_), C–S–H gel, and C–A–H gel. The characteristic peak intensity of calcium hydroxide is relatively high, indicating that it is present in significant quantities in the system and is a potentially important component for carbonation. After the addition of mineral admixtures, the types of hydration products in the system increased significantly, and distinct quartz characteristic peaks were observed, indicating that the siliceous components in the admixtures did not fully participate in the reaction. In addition, diffraction reflections attributable to hydrotalcite-like phases, including meixnerite and hydrotalcite, were identified in the multicomponent systems, indicating the formation of Mg–Al-bearing layered phases during hydration. Owing to their layered structure and anion-exchange characteristics, these phases may interact with carbonate species and contribute to the regulation of alkalinity during subsequent carbonation. Concurrently, some calcium aluminate hydrates and zeolite-like minerals were also formed within the composite cementitious material system. The formation of these hydration products reflects the complex reaction processes within the multicomponent system, and their structural characteristics may influence the system’s density and subsequent carbonation behavior. Notably, characteristic peaks of calcite (CaCO_3_) were observed in all samples, indicating that carbonation reactions had occurred to varying degrees during the hydration process. In samples with lower OPC content, the characteristic peak of calcium hydroxide was significantly weakened or even disappeared; this may be due to part of it being consumed by the pozzolanic reaction, while another part underwent carbonation during hydration and converted into calcium carbonate. In the composite cementitious material system, calcium hydroxide and some gel products serve as the primary carbonatable components, while hydrotalcite-like phases may participate in carbonation-related ion-exchange and phase-transformation processes. These phase characteristics provide a basis for interpreting the subsequent carbonation behavior and CO_2_ sequestration response of the multicomponent system.

(2) XRD Analysis After Carbonation

**Figure 13:**
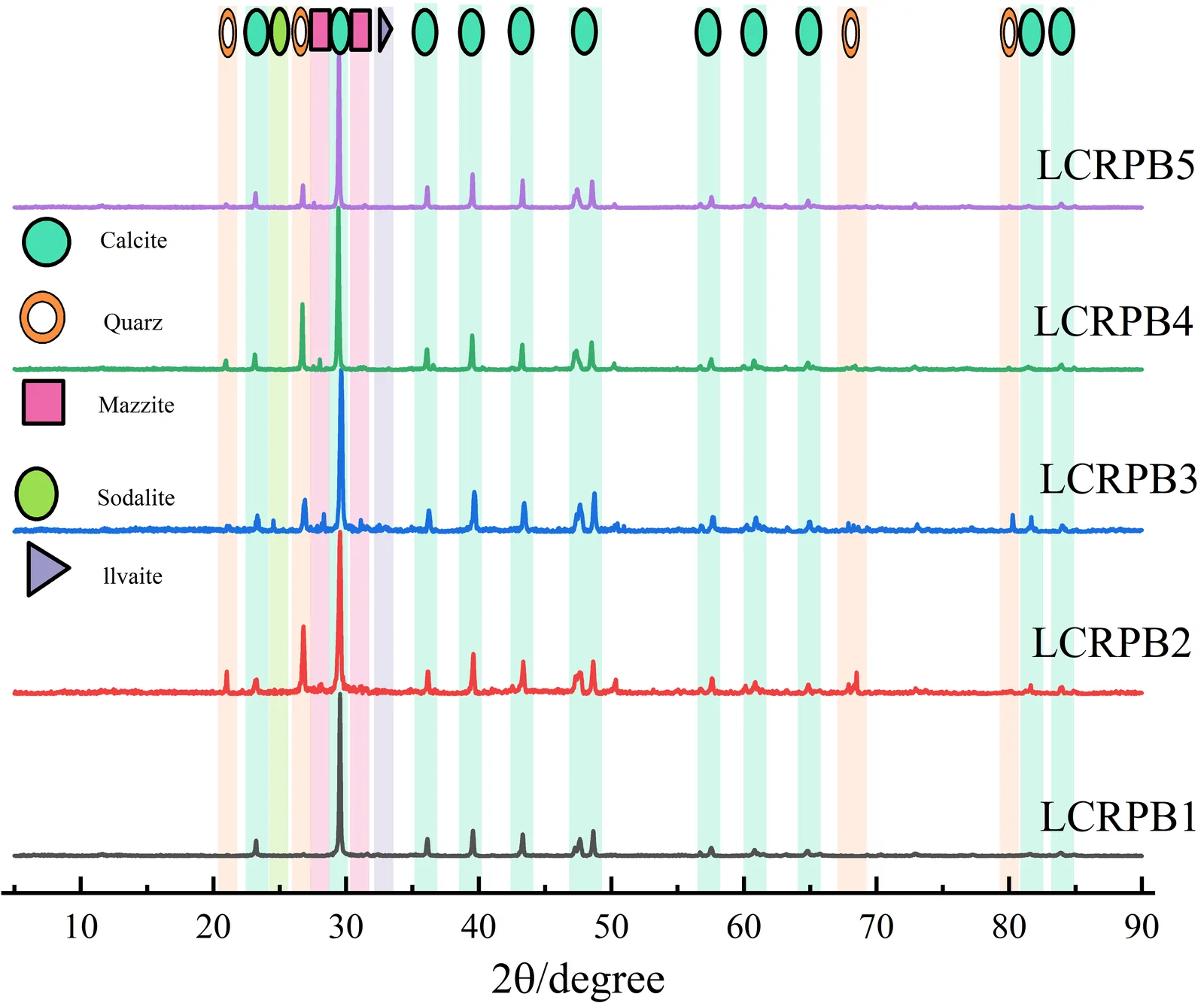

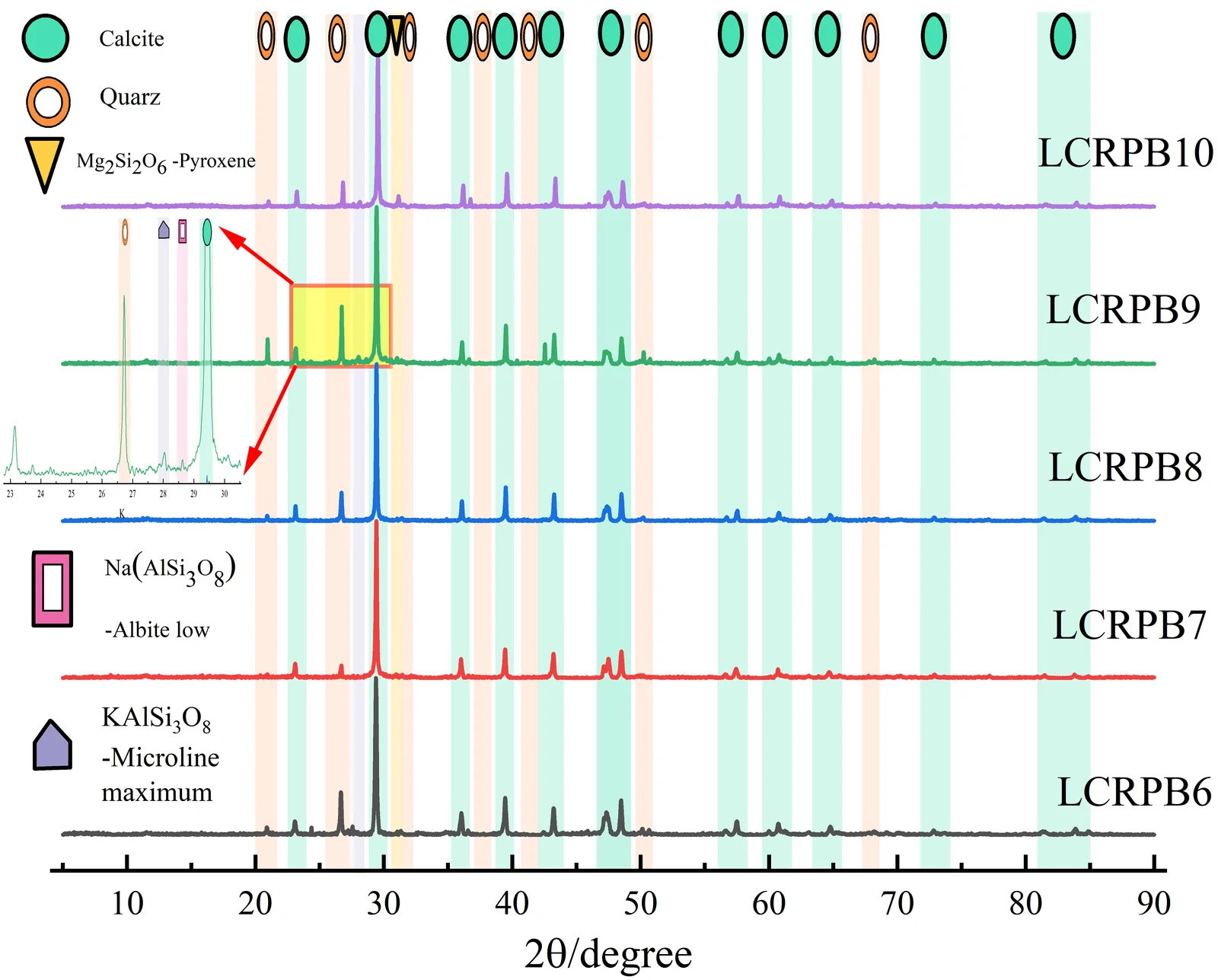

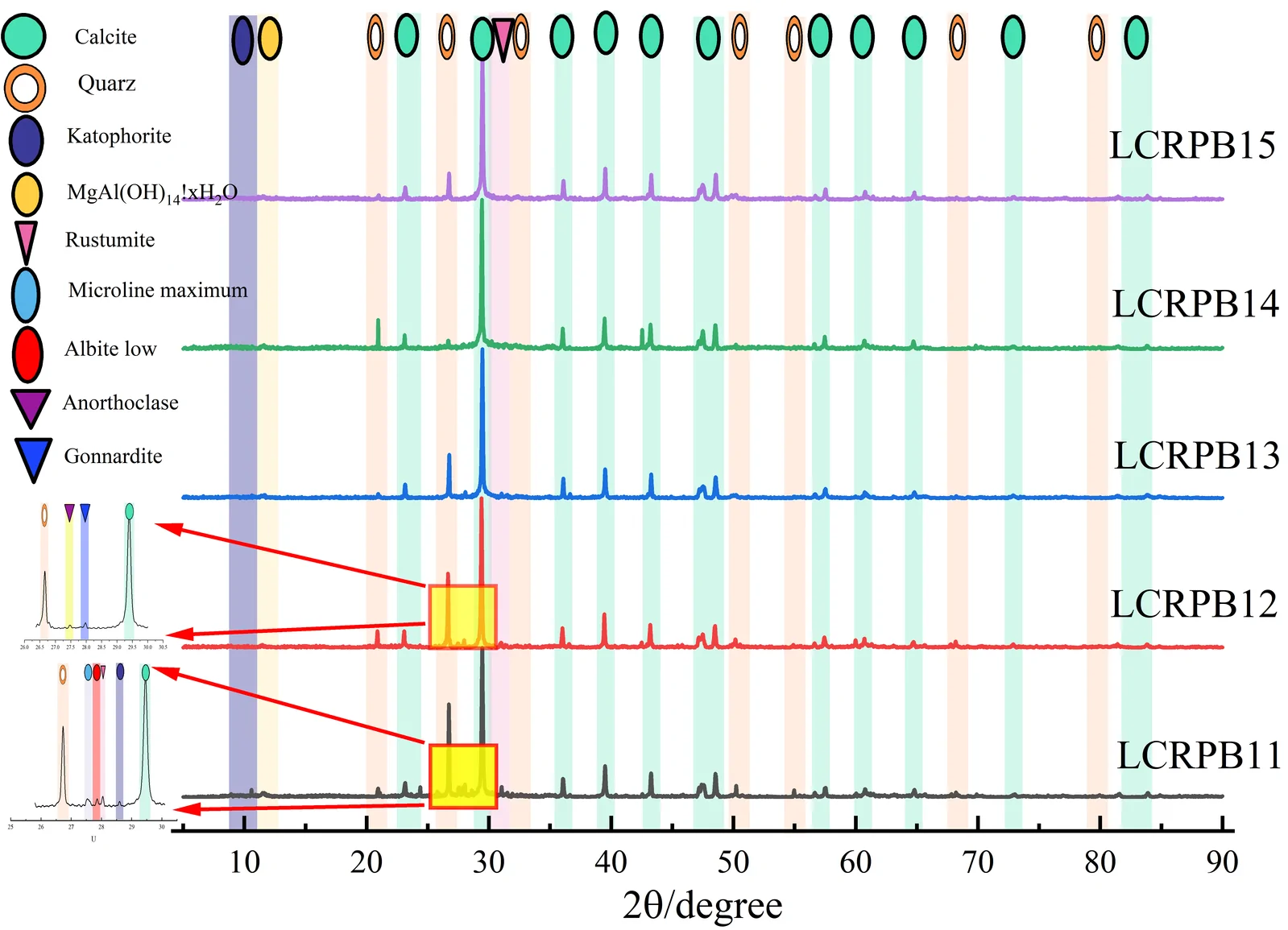

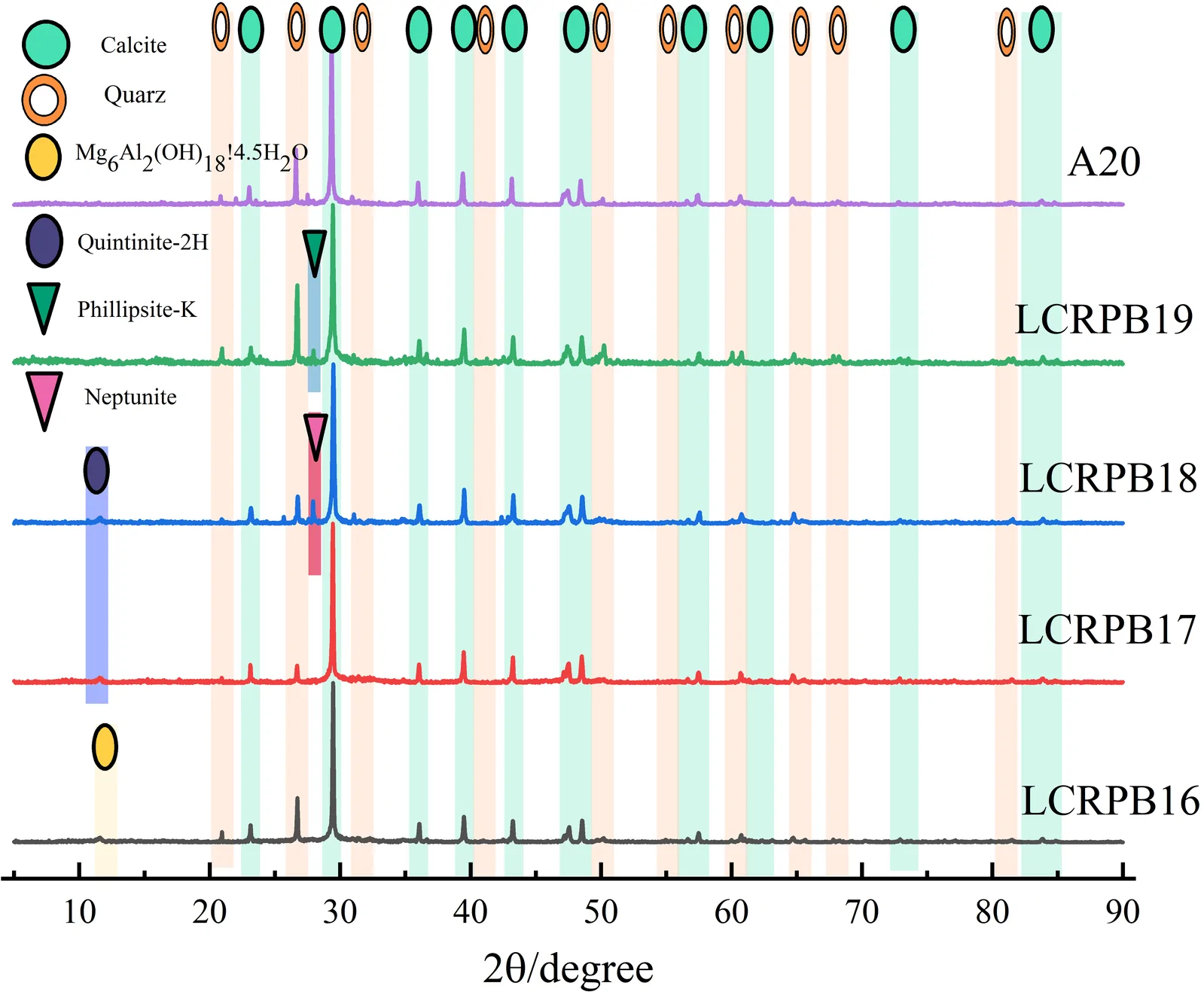

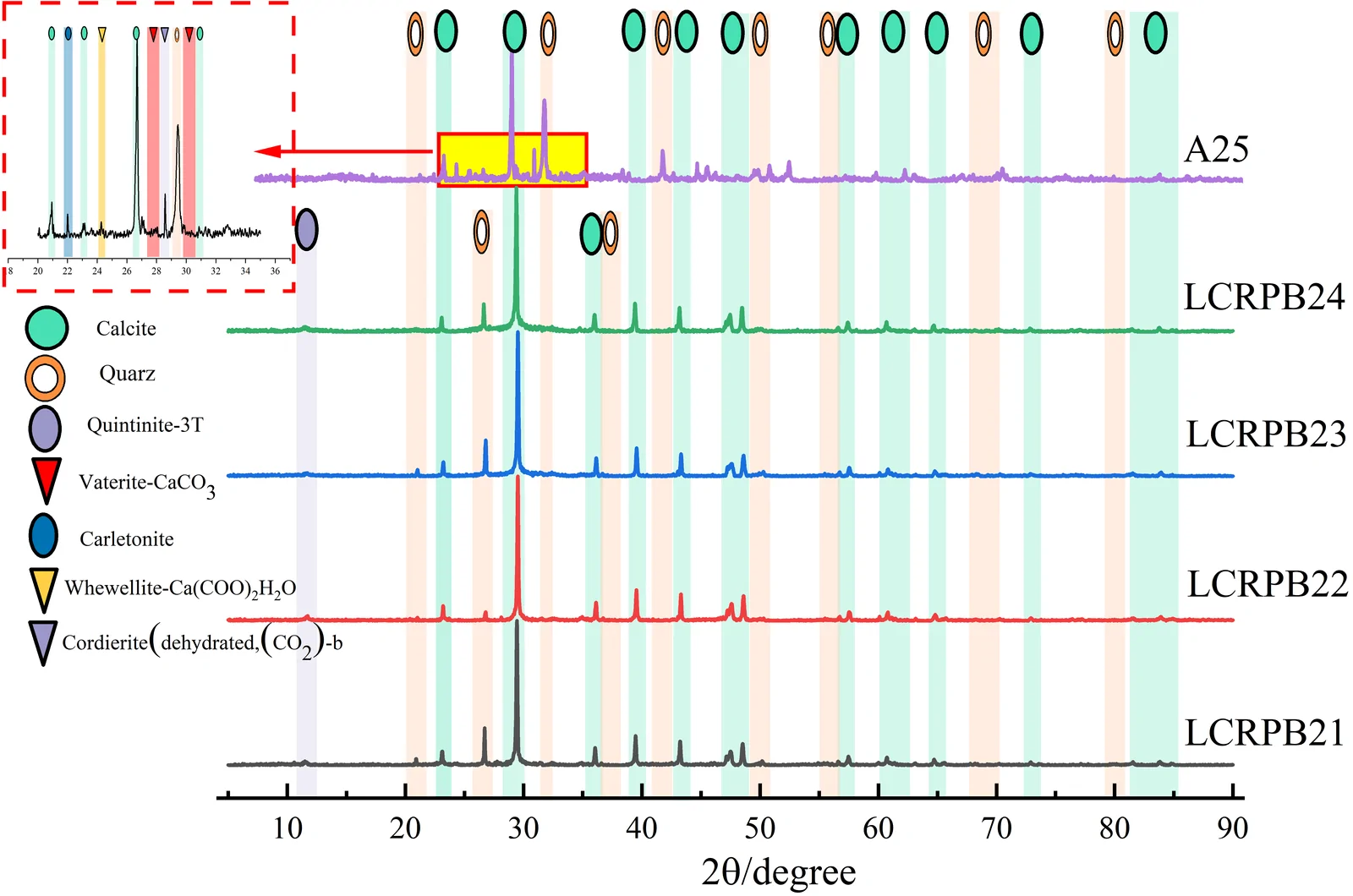
XRD spectrum of carbonated LCRPB.

The gel-forming materials from each group were hydrated for 28 days and then carbonated using a wet method until the pH reached 7. The XRD results are shown in Figure 13. As shown in the figure, the main product in all systems after carbonation is calcium carbonate (CaCO_3_), accompanied by a small amount of unreacted quartz phase. Compared with the hydration products before carbonation, the characteristic peaks of calcium hydroxide and most gel products have significantly weakened or even disappeared, indicating that the main carbonizable components in the system substantially participated in the reaction during the carbonation process. In the pure cement system, the carbonation products are predominantly calcium carbonate. Combined with the pre-carbonation XRD results, it is evident that hydration products such as calcium hydroxide, C-S-H, and C-A-H all participated in the carbonation reaction. Among these, the low-intensity broad peaks observed in the XRD patterns may correspond to amorphous silica gel formed after decalcification of the gel products. For composite cementitious material systems incorporating mineral admixtures, the post-carbonation composition consists primarily of calcium carbonate and unreacted siliceous minerals. This indicates that some inert or low-reactivity components in the admixtures did not react during carbonation, while the main hydration products were converted into calcium carbonate during the process. Furthermore, the characteristic peaks of calcium hydroxide have largely disappeared in all sample groups, indicating that it preferentially participates in the reaction during carbonation, which is consistent with previous research findings. The changes observed in the diffraction features associated with hydrotalcite-like phases after carbonation are consistent with their participation in carbonation-related phase transformations and suggest possible interactions with carbonate species during the wet-carbonation process. Such behavior may contribute to the evolution of alkalinity and phase composition during carbonation.

During the wet carbonation process, the observed increase in crystalline carbonate phases indicates substantial carbonation under the present accelerated conditions, indicating that this method can effectively achieve the carbonation reaction of the system. Therefore, the change in CaCO_3_ content before and after carbonation was used to estimate the CO_2_ sequestration potential of LCRPB. Accordingly, the present phase analysis is used primarily to support the qualitative interpretation of carbonation pathways, while the relative contributions of individual carbonatable phases to CO_2_ uptake require further quantitative clarification.

### 3.6. Carbon Sequestration Potential

**Figure 14:**
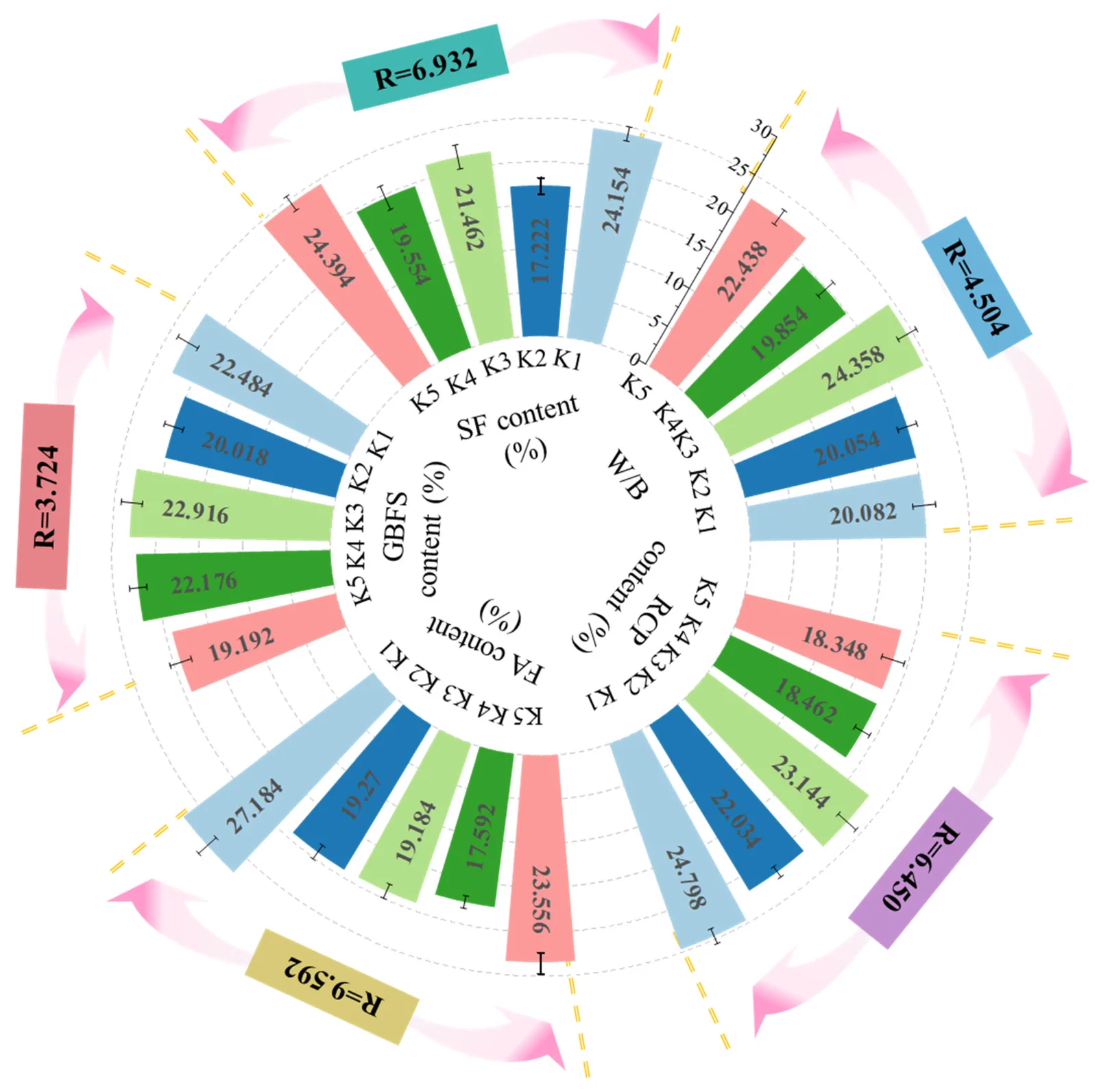
LCRPB’s carbon sequestration capacity.

The carbon sequestration potential of LCRPB was calculated using Equations (1) and (2) in Section 2.4, and the effects of various factors on the CO_2_ sequestration capacity of the cementitious material are shown in Figure 14. Overall, the CO_2_ sequestration capacity varied across the investigated factor levels, exhibiting composition-dependent response patterns. As the water-to-binder ratio increases, the carbon sequestration capacity of the cementitious material first increases and then decreases. This trend is generally consistent with the change in calcium hydroxide content in the system after 28 days of hydration, suggesting that calcium hydroxide, as one of the primary carbonatable phases, has an important influence on the system’s CO_2_ sequestration capacity. As the RCP content increases, the carbon sequestration capacity of the cementitious material shows an overall downward trend. This is primarily due to the low reactivity of RCP, whose incorporation dilutes the cement hydration products, thereby reducing the effective components available for carbonation reactions in the system and leading to a decrease in carbon sequestration capacity. For GBFS, the carbon sequestration capacity of the cementitious material fluctuates within a narrow range as the content increases. This indicates that GBFS exerts a dual effect on the system’s carbon sequestration capacity: on the one hand, its high reactivity consumes calcium hydroxide and generates gel-like products; on the other hand, its high calcium content may provide a potential source for CO_2_ fixation. As the SF admixture content increases, the carbonation capacity of the cementitious material first decreases and then increases. At higher admixture levels, its carbonation capacity even exceeds that of the system without SF. This may be related to SF’s high pozzolanic activity and its fine-particle effect, which promote hydration reactions and increase the content of potential carbonizable components in the system. An increase in FA content causes the carbon sequestration capacity of the cementitious material to first decrease, then stabilize, and finally increase. Compared to SF and GBFS, FA has lower reactivity; initially, it primarily exerts a dilution effect, leading to a reduction in carbonizable substances. However, at higher content levels, its particle morphology and dispersion effects may promote the hydration process to some extent, thereby improving carbon sequestration capacity. The range analysis results (Figure 14) showed that the relative influence of the investigated factors on CO_2_ sequestration capacity followed the order FA content > SF content > RCP content > W/B > GBFS content (D > B > E > A > C). Based on the level-wise responses, A3B5C3D1E1 was identified as the factor-level combination associated with the highest CO_2_ sequestration capacity. ANOVA results (Table 10) showed that the individual factor effects did not reach statistical significance. The composition-dependent variations in CO_2_ sequestration capacity were therefore further examined in relation to hydration-derived carbonatable phases and the characteristics of the subsequent carbonation process.

However, the underlying causes of the differences in carbon sequestration capacity among the various mixing ratio systems remain unclear; these differences are not only related to material composition but may also be closely linked to the carbonation reaction process and its kinetic characteristics. Therefore, it is necessary to conduct a further in-depth analysis of the formation mechanism from the perspective of carbonation behavior.

### 3.7. Characteristics of the Carbonation Reaction and Their Impact on Carbon Sequestration Capacity

**Figure 15:**
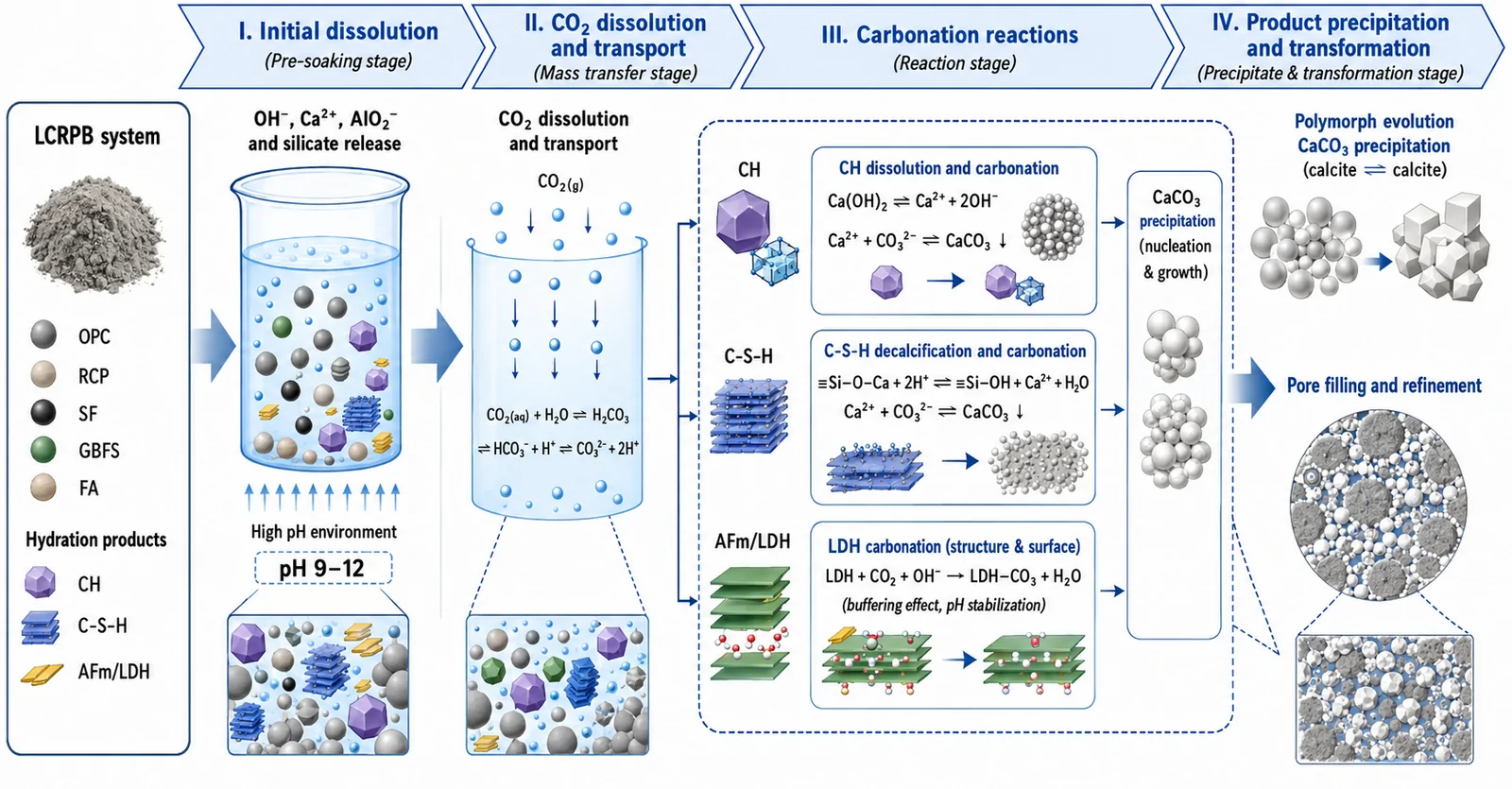
Schematic diagram of the wet carbonation reaction mechanism in the LCRPB system.

To elucidate the mechanisms underlying the differences in carbon sequestration capacity among composite cementitious materials with varying compositions, this study analyzes the characteristics of the wet carbonation reaction process. The overall wet carbonation mechanism of the LCRPB system is illustrated in Figure 15, where the reaction process can be divided into four key stages: alkaline ion leaching, CO_2_ dissolution and ionization, carbonatable phase reaction, and dynamic pH evolution. In hydrated LCRPB, Ca(OH)_2_ and other calcium-bearing hydration products provide sufficient alkaline reserve and reactive phases for subsequent carbonation. Moreover, hydrotalcite-like LDH phases can participate in wet carbonation owing to their special layered structure and anion-exchange ability. The continuous dissolution of alkaline components and progressive decalcification/carbonation of hydration products jointly reduce the solution alkalinity, while the existence of LDH phases further regulates pH variation through carbonate-related ion exchange. Accordingly, the differences in pH evolution among various LCRPB mixtures are comprehensively determined by hydration product assemblage, initial alkalinity reserve, and the content of carbonatable phases.

**Figure 16:**
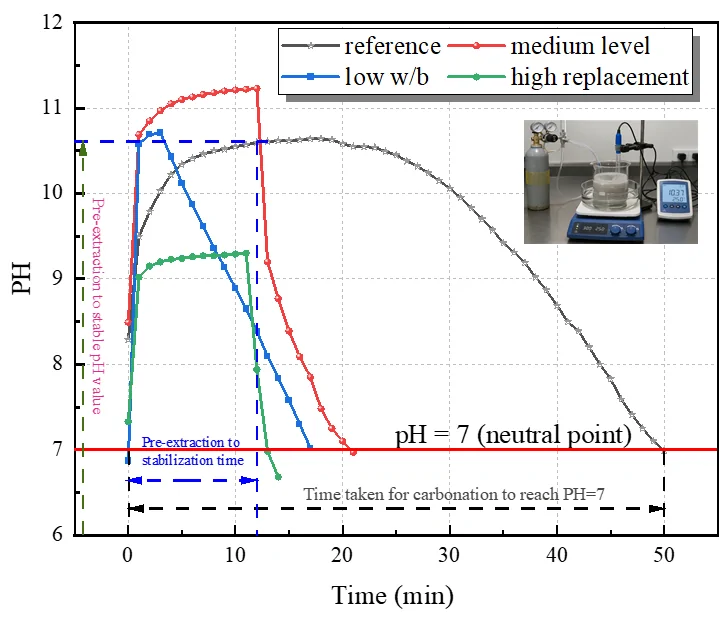
pH changes during the wet carbonation of microfine powder in orthogonal test slurry specimens.

Based on the above mechanism, the pH evolution characteristics during wet carbonation are further analyzed, as shown in Figure 16. In this experiment, powders obtained by grinding 28-day hydrated net paste specimens were mixed with water at a liquid-to-solid ratio of 5 and pre-leached until the pH value stabilized. Subsequently, CO_2_ was introduced at a gas flow rate of 3 L/min to initiate the wet carbonation reaction. The different LCRPB formulations exhibited distinct wet carbonation behaviors in terms of pre-leaching stabilization time, stable pH value, pH decline rate, and neutralization time (pH = 7). These characteristic parameters effectively reflect the alkaline leaching behavior, initial alkalinity level, and dynamic carbonation kinetics, providing essential indicators for evaluating the carbonation performance and CO_2_ sequestration potential of LCRPB. Therefore, pre-leaching stabilization time, equilibrium pH, pH decline rate, and time required to reach pH = 7 were selected as core indices to characterize the overall wet carbonation process.Based on the above analysis, the following discussion systematically investigates the effects of multiple factors on the wet carbonation behavior and CO_2_ sequestration capacity of LCRPB, focusing on four key parameters: pre-leaching stabilization time, pre-leaching stabilization pH, pH decline rate, and carbonation neutralization time.

#### 3.7.1. Pre-Leaching Stabilization Behavior

**Figure 17:**
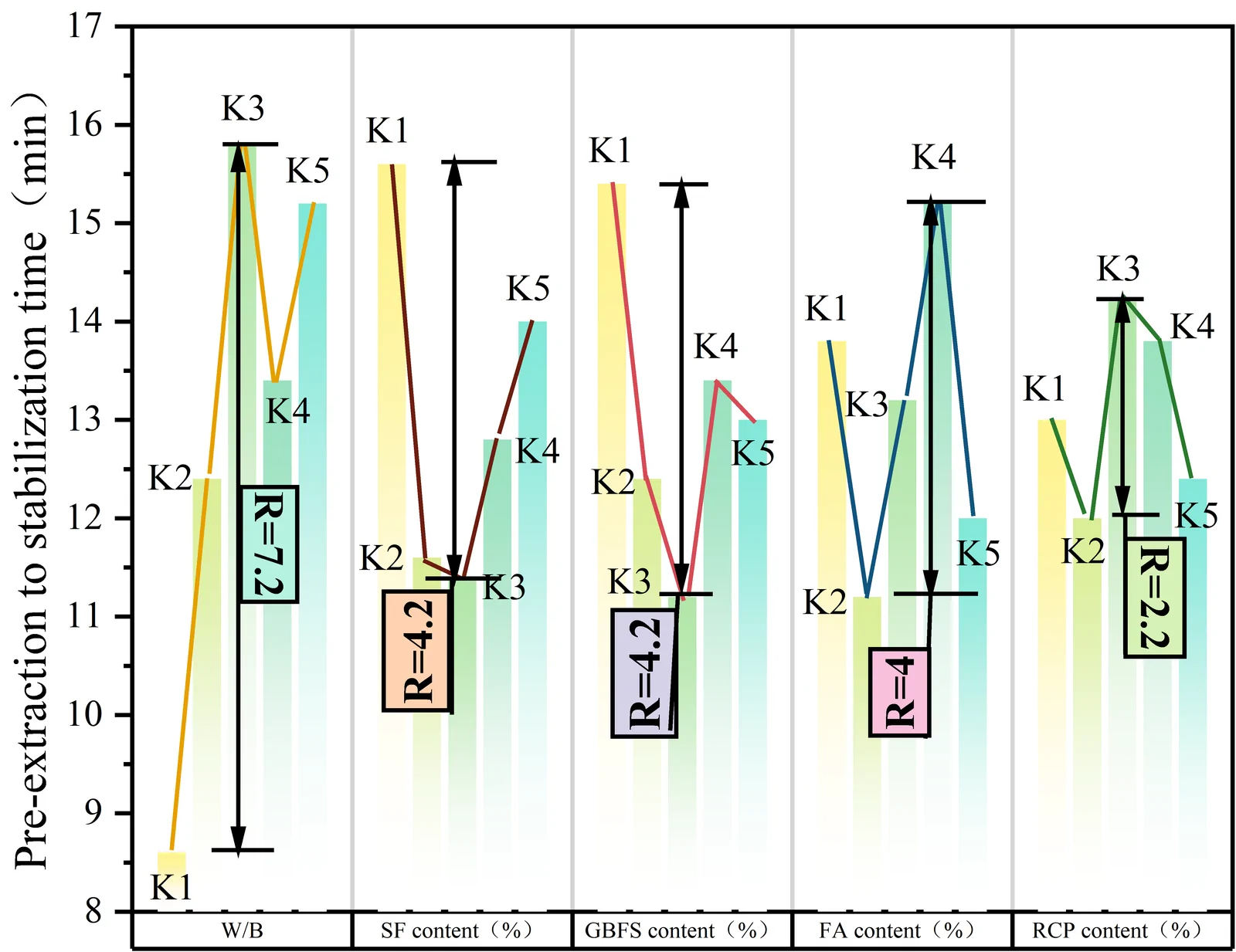
Effect of variables in the orthogonal experiment on the time required for pre-leaching stabilization.

The time required for pre-leaching to reach a stable state reflects the progressive establishment of dissolution–equilibrium conditions for soluble alkaline species prior to carbonation. As shown in Figure 17, the time from pre-leaching to stabilization exhibits a “first-rise-then-decline” trend as the water-to-binder ratio increases: within the low-to-medium water-to-binder ratio range (Levels 1–3), the time increases from 8.6 min to 15.8 min, indicating that, as the degree of hydration increases and soluble components rise, the time required for the system to reach dissolution-diffusion equilibrium is prolonged; when the water-to-binder ratio further increases (Levels 3–5), the time decreases and fluctuates between 13 and 15 min, indicating that increased porosity and accelerated diffusion dominate, leading to a faster establishment of dissolution and equilibrium. For mineral admixtures, GBFS, FA, RCP, and SF all exhibit a “decrease followed by an increase” pattern: when added in small amounts, the volcanic ash reaction consumes Ca(OH)_2_ and forms a denser C–S–H gel, reducing ion solubility and thereby shortening the stabilization time; as the admixture content continues to increase, Ca(OH)_2_ is consumed in large quantities and the structure becomes more complex, inhibiting diffusion and leading to an increase in the time required to reach stability. Range analysis showed that the relative influence of the investigated factors on the pre-leaching stabilization time followed the order W/B > SF content ≈ GBFS content > FA content > RCP content (A > B ≈ C > D > E), with W/B exhibiting the largest response variation. ANOVA results (in Table 11) showed no statistically significant individual factor effects. The observed variation in stabilization time reflects the combined response of hydration state, alkaline-component availability, and ion-transport characteristics during the pre-leaching stage.

#### 3.7.2. Pre-Leaching Equilibrium pH

**Figure 18:**
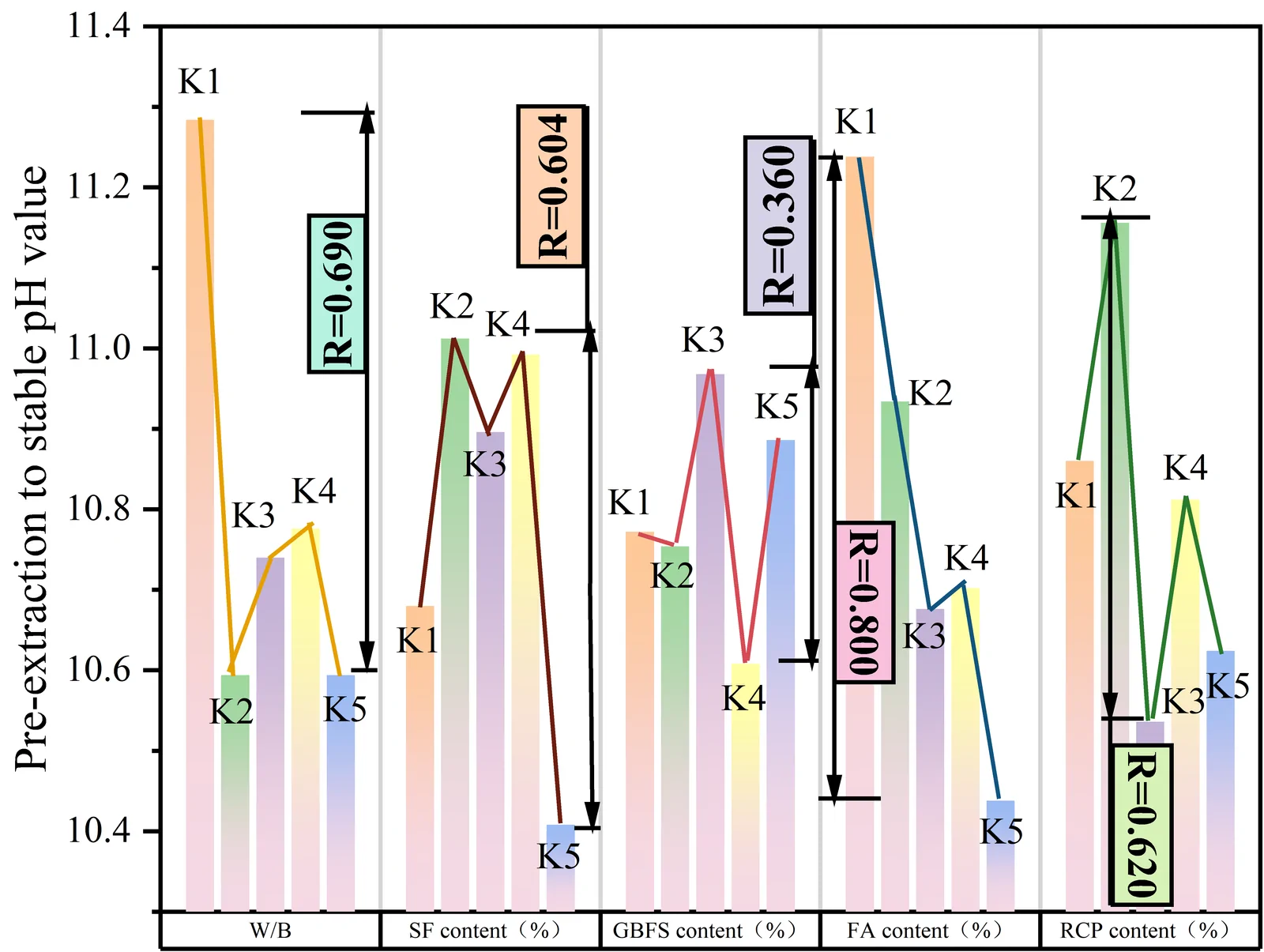
Effect of variables in an orthogonal experiment on the stable pH value during powder pre-leaching.

The pre-leaching equilibrium pH reflects the initial alkalinity and alkaline reserve of the system, which influences CO_2_ absorption potential and carbonation kinetics. As shown in Figure 18, the equilibrium pH first decreased and then gradually stabilized with increasing water-to-binder ratio. This is mainly because a higher water-to-binder ratio promotes cement hydration and pozzolanic reactions, accelerating the consumption of Ca(OH)_2_ while diluting the OH^−^ concentration in solution. At higher water-to-binder ratios, the hydration-promoting effect gradually weakened, and the generation and consumption of Ca(OH)_2_ approached dynamic equilibrium, resulting in a stable pH.

For LCRPB, the equilibrium pH initially increased and then decreased with increasing admixture content. At low dosages, unhydrated active components in RCP and the nucleation effect of fine particles promoted cement hydration, increasing the production of Ca(OH)_2_ and alkaline ions. Although GBFS and SF consumed part of the Ca(OH)_2_, their early hydration-promoting effects remained dominant. With further increases in admixture content, pozzolanic reactions became more significant, consuming larger amounts of Ca(OH)_2_, while the denser gel structure restricted ion release, leading to reduced alkalinity. In contrast, increasing FA content continuously decreased the equilibrium pH due to its low early-stage reactivity and dilution effect. Range analysis showed that the relative influence of the investigated factors on the pre-leaching equilibrium pH followed the order FA content > W/B > RCP content > SF content > GBFS content (D > A > E > B > C). ANOVA results (Table 12) showed no statistically significant individual factor effects. Nevertheless, the equilibrium-pH responses exhibited clear composition-dependent variations associated with differences in hydration degree, alkaline reserve, and the release of soluble alkaline species. Overall, the pH value of the pre-leached suspension reflects the comprehensive balance between the system’s alkalinity reserve, the release of alkaline components, and their consumption through reactions with pozzolanic materials. Higher initial alkalinity facilitates the dissolution and release of carbonatable components; however, both excessively high and excessively low alkalinity may affect the dissolution of CO_2_ in the liquid phase and the subsequent carbonation reaction process.

#### 3.7.3. Kinetics of the Carbonation Reaction of LCRPB

**Figure 19:**
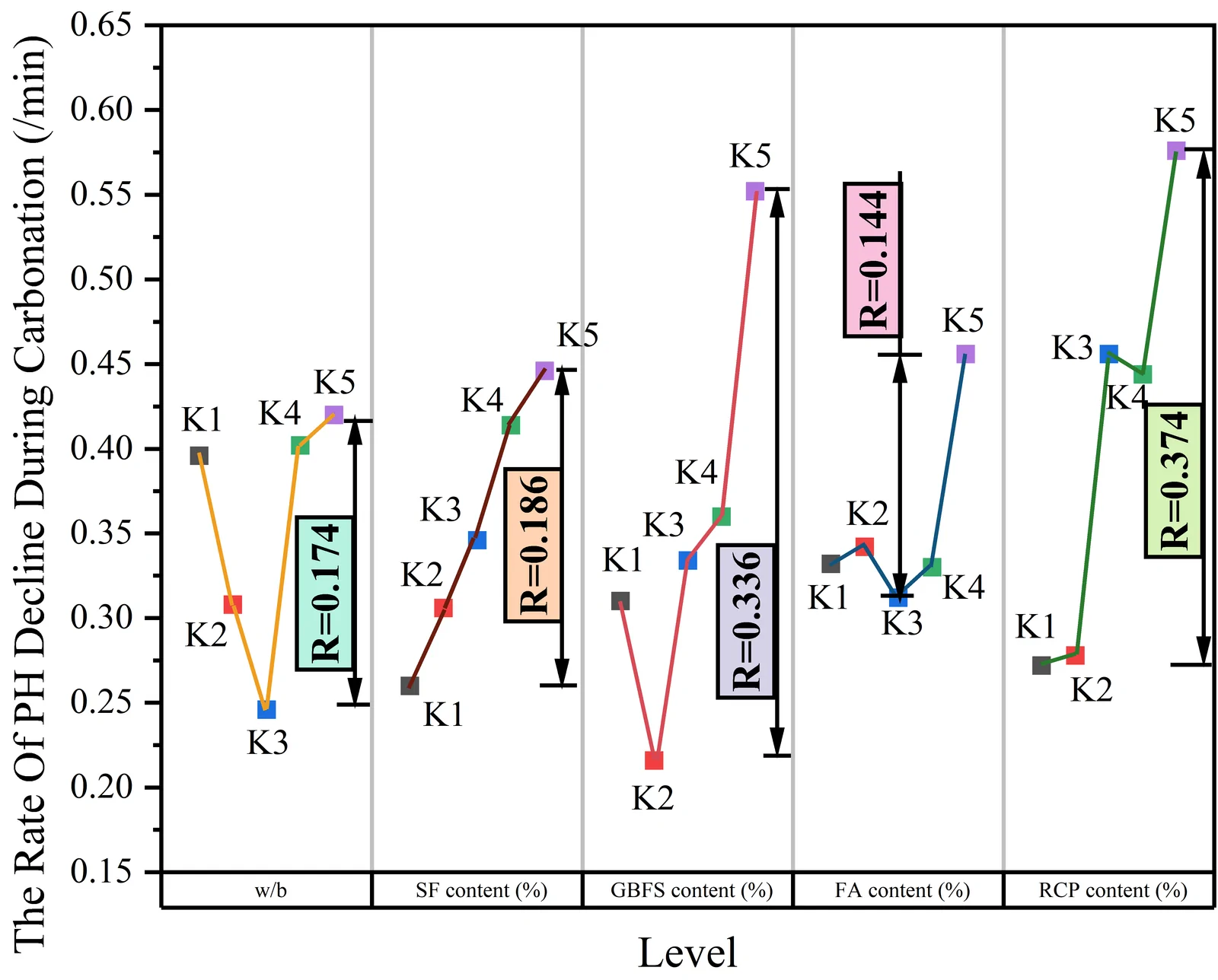
Effect of variables in the orthogonal experiment on the rate of pH decline during the powder carbonation process.

Figure 19 shows the effects of various variables in the orthogonal experiment on the rate of pH decline during the carbonation process of the powder. As the water-to-binder ratio increases, the pH decline rate first decreases and then increases. This is because an increased water-to-binder ratio enhances the degree of hydration, leading to a greater amount of hydration products available for carbonation in the system, which slows down the pH decline rate. However, when the water-to-binder ratio increases further, its effect on the degree of hydration becomes negligible; instead, it introduces more pores, allowing for more thorough contact between the hydration products and carbon dioxide during carbonation, thereby accelerating both the reaction rate and the pH decline rate. Increased dosages of both RCP and GBFS cause the pH decline rate to first decrease and then increase. This is consistent with the effects of various mineral admixtures on the pre-leaching stable pH value discussed earlier. The addition of an appropriate amount of mineral admixtures promotes cement hydration, thereby increasing the quantity of hydration products and reducing the pH decline rate. However, the use of excessive mineral admixtures consumes a large amount of calcium hydroxide, reducing the pH buffering capacity and consequently accelerating the pH decline rate. A noticeable increase in the pH decline rate was observed only when the FA content reached the fourth level, which may be related to its relatively low pozzolanic activity. The relative response patterns obtained from the range analysis and ANOVA were broadly consistent, with RCP and GBFS exhibiting comparatively greater variations in the pH decline rate. Although the individual factor effects did not reach statistical significance (Table 13), the observed response characteristics indicate that the carbonation kinetics are governed by the coupled evolution of hydration products, alkaline buffering capacity, and pore-mediated CO_2_ transport.

#### 3.7.4. Time Required to Reach the pH = 7 Operational Endpoint

**Figure 20:**
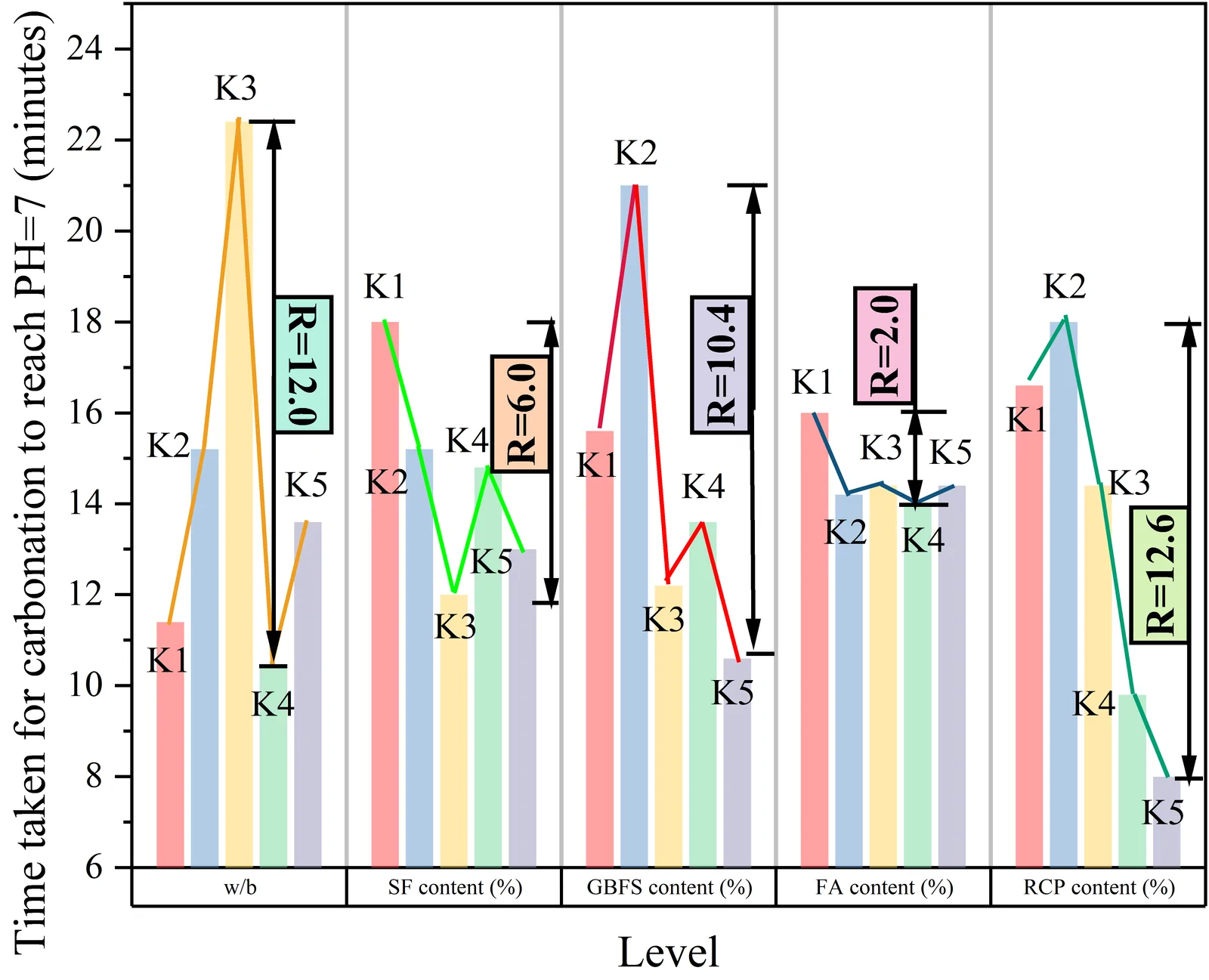
Effect of variables on the time required for carbonation to reach pH = 7.

Figure 20 presents the effects of different factors on the time required for carbonation to reach pH = 7. This parameter characterizes the duration of the wet-carbonation process under the present test conditions and is influenced by the type and amount of hydration products. As the water-to-binder ratio increased, the carbonation time first increased and then decreased. A moderate increase in water content promoted hydration and increased the amount of carbonatable phases, thereby prolonging carbonation. However, excessive water accelerated pozzolanic reactions, reduced Ca(OH)_2_ content, and increased pore volume, resulting in faster carbonation. With increasing RCP content, the carbonation time also exhibited a trend of first increasing and then decreasing. At low dosages, RCP promoted hydration and provided additional carbonatable components, while excessive RCP introduced a dominant dilution effect that accelerated carbonation. The influence of GBFS differed noticeably from that of SF and FA. Increasing SF and FA contents gradually shortened the carbonation time, whereas GBFS produced a more pronounced prolongation of carbonation at moderate dosages. This is mainly because FA has relatively low pozzolanic activity and limited influence on carbonation, while SF forms low-Ca/Si gel products that contribute less to carbonation. In contrast, GBFS contains a high Ca content, and its hydration products provide more carbonatable phases, thereby extending the carbonation process. Range analysis showed that the relative influence of the investigated factors on the time required to reach pH = 7 followed the order RCP content > W/B > GBFS content > SF content > FA content (E > A > C > B > D). ANOVA results (Table 14) identified RCP content as a statistically significant factor, highlighting its important role in regulating the duration of the wet-carbonation process. The time required to reach the predefined pH = 7 endpoint therefore provides a useful kinetic parameter for characterizing the carbonation response of LCRPB under the present accelerated test conditions.

### 3.8. Recycling

**Figure 21:**
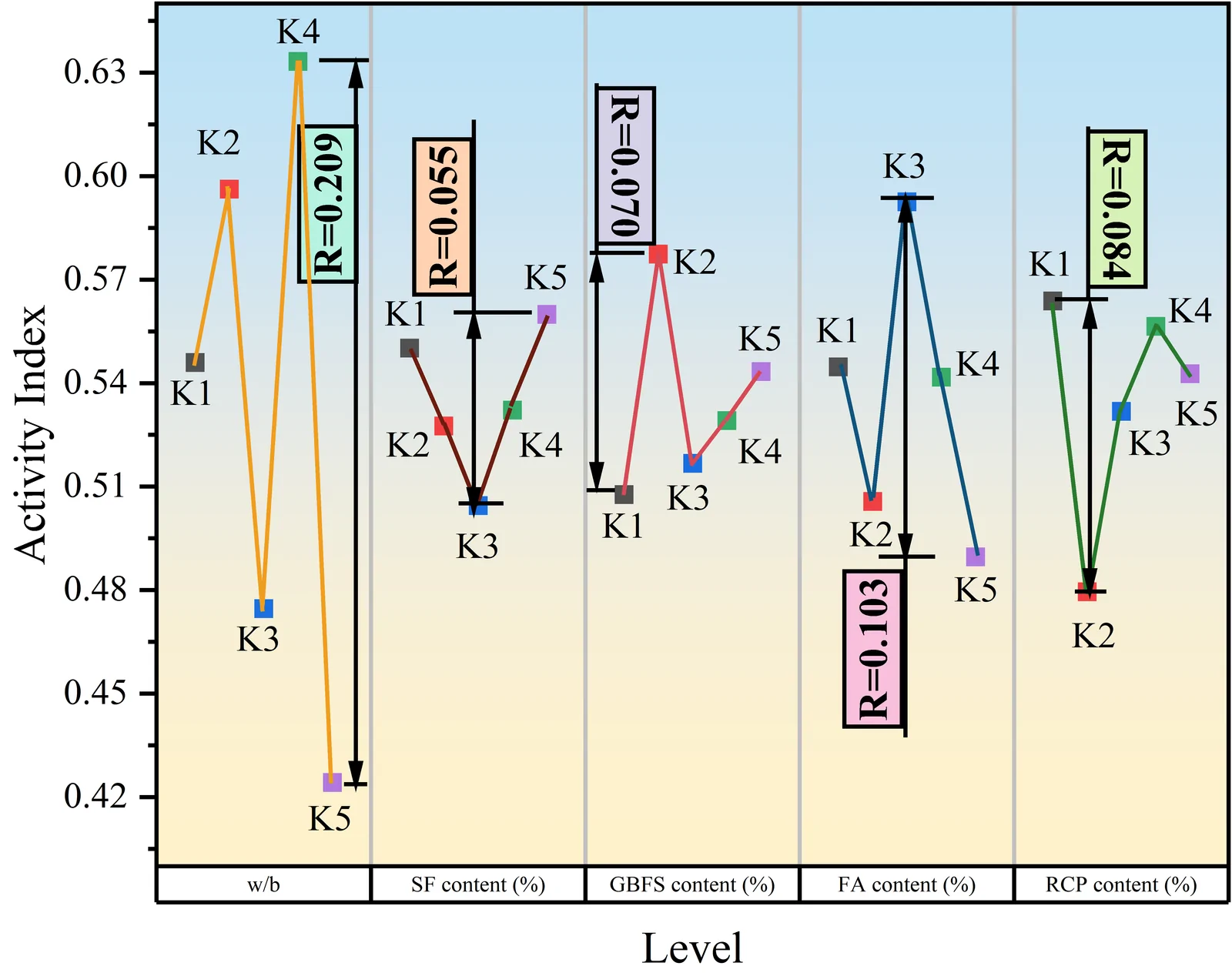
Effect of Factors in an Orthogonal Experiment on the Activity Index of Cementitious Materials After Hydration and Carbonation.

Figure 21 illustrates the variation in the activity index of carbonated powders under different factor levels. As the water-to-binder ratio increased, the activity index fluctuated and reached its highest value at the fourth level, while comparable variations were also observed for the mineral admixtures. These trends may be related to differences in hydration degree, CaCO_3_ formation during carbonation, and the proportion of residual reactive components. An appropriate amount of CaCO_3_ may improve particle packing and provide nucleation sites for subsequent hydration, whereas excessive carbonate formation may increase the proportion of relatively inert phases.

Range analysis showed that the relative variation in the post-carbonation activity index followed the order W/B > FA content > RCP content > GBFS content > SF content (A > D > E > C > B). ANOVA results (Table 15) showed no statistically significant individual factor effects, while the activity index remained within a comparable range across the investigated factor levels. The residual activity of carbonated LCRPB therefore reflects the combined effects of hydration history, carbonate formation, and the remaining reactive phases in the multicomponent binder system. Overall, the retained activity of the carbonated powders supports their potential for subsequent utilization in cement-based materials.

### 3.9. Integrated Discussion of Hydration, Carbonation, and Reuse

The engineering and carbonation behaviors of LCRPB are closely linked through hydration and phase evolution. Variations in W/B and the proportions of SF, GBFS, FA, and RCP alter the balance among dilution, reactivity, and particle-packing effects, thereby affecting hydration development and mechanical performance. In particular, increasing RCP content reduces hydration heat because of its relatively low reactivity and the progressive replacement of OPC, whereas appropriate incorporation of supplementary cementitious materials can promote hydration and improve strength through secondary reactions and particle-packing effects.

Hydration development further determines the phase assemblage and alkalinity available for subsequent carbonation. Ca(OH)_2_ and other hydration products provide the principal carbonatable constituents, while differences in phase composition and alkaline reserve are reflected in pre-leaching behavior, pH evolution, and carbonation kinetics. The increased formation of CaCO_3_ identified by XRD and TGA/DTG confirms the transformation of these carbonatable phases during wet carbonation and provides the basis for the observed CO_2_ sequestration. Because the present phase characterization is primarily qualitative or semi-quantitative, the contribution of individual phases to CO_2_ uptake cannot be quantitatively distinguished.

After carbonation, the powder retained measurable residual activity, indicating that CO_2_ mineralization does not eliminate its reuse potential. The post-carbonation response is likely governed by the combined effects of residual reactive constituents and carbonation-induced phase transformation rather than by a single compositional factor. Overall, the results establish a coherent composition–hydration–carbonation–reuse framework, which provides the mechanistic basis for the subsequent multi-objective optimization of engineering performance, CO_2_ sequestration, and post-carbonation activity.

### 3.10. Multi-Objective Selection of a Balanced Formulation Considering Engineering Performance, Carbon Sequestration Potential, and Recyclability

To comprehensively integrate the engineering performance, wet-carbonation behavior, CO_2_ sequestration performance, and post-carbonation recyclability of LCRPB, the matrix analysis method for orthogonal experiments proposed by Zhou et al. [45] was employed to evaluate the combined effects of different factor levels on multiple performance indicators. The method constructs three matrices, namely the response matrix M, normalization matrix T, and factor-effect matrix S, to determine the contribution of each factor level to a given evaluation indicator. For an orthogonal experiment containing l factors with m levels for each factor, $k_{ij}$ denotes the mean response of a given evaluation indicator at the jth level of factor $A_{i}$. To ensure a consistent preference direction among different evaluation indicators, the transformed response $K_{ij}$ was defined as Equation (7):

(7) $$K_{ij}=\left\{\begin{array}{c} k_{ij}\;for\;benefit-type\;indicators\\ \frac{1}{k_{ij}}\;for\cos t-type\;indicators \end{array}\right.$$

Here, benefit-type indicators refer to those for which a larger value is preferred, whereas cost-type indicators refer to those for which a smaller value is preferred. Based on the transformed responses, the response matrix M was constructed as follows:

$$M=\left[\begin{array}{ccccc} K_{11} & 0 & 0 & \text{…} & 0\\ K_{12} & 0 & 0 & \text{…} & 0\\ \text{…} & \text{…} & \text{…} & \text{…} & \text{…}\\ K_{1m} & 0 & 0 & \text{…} & 0\\ 0 & K_{21} & 0 & \text{…} & 0\\ 0 & K_{22} & 0 & \text{…} & 0\\ \text{…} & \text{…} & \text{…} & \text{…} & \text{…}\\ 0 & K_{2m} & 0 & \text{…} & 0\\ \text{…} & \text{…} & \text{…} & \text{…} & \text{…}\\ 0 & 0 & 0 & \text{…} & K_{l1}\\ 0 & 0 & 0 & \text{…} & K_{l2}\\ \text{…} & \text{…} & \text{…} & \text{…} & \text{…}\\ 0 & 0 & 0 & \text{…} & K_{\mathrm{lm}} \end{array}\right]$$

For each factor $A_{i}$ the normalization coefficient $T_{i}$ was calculated as Equation (8):

(8) $$T_{i}=\frac{1}{\sum_{j=1}^{m}K_{ij}},\;i=1,2,\ldots ,l.$$

Accordingly, the normalization matrix T was expressed as follows:

$$T=\left[\begin{array}{cccc} T_{1} & & & \\ & T_{2} & & \\ & & \ldots & \\ & & & T_{l} \end{array}\right]$$

To account for the relative magnitude of the effect of each factor, $S_{i}$ was defined as the range of factor $A_{i}$ for the corresponding evaluation indicator. The normalized factor-effect coefficient $S_{i}$ was calculated as Equation (9):

(9) $$S_{i}=\frac{s_{i}}{\sum_{i=1}^{l}s_{i}},\;i=1,2,\ldots ,l.$$

The factor-effect matrix S was then expressed as

$$S=\left[\begin{array}{c} S_{1}\\ S_{2}\\ \ldots \\ S_{l} \end{array}\right]$$

The weight vector corresponding to a given evaluation indicator was subsequently calculated using Equation (10):

(10) ω=MTS

In Equation (10), the product MT represents the normalized contribution of each factor level to the corresponding evaluation indicator, whereas S incorporates the relative magnitude of the factor effect determined from range analysis. Therefore, the resulting weight vector ω reflects the comprehensive contribution of each factor level to a given evaluation indicator.

**Figure 22:**
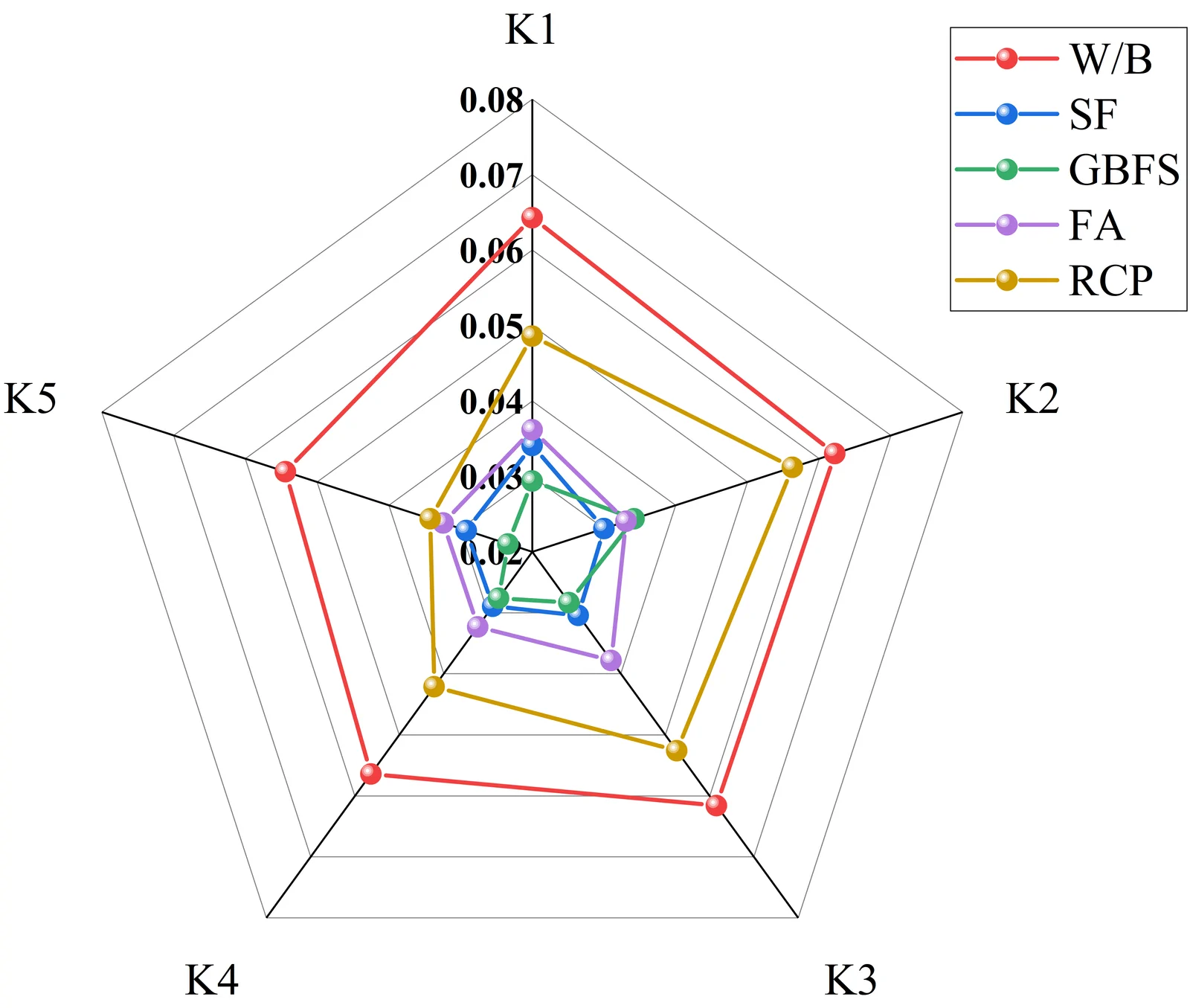
Comprehensive influence weights of different factor levels on the overall performance of LCRPB.

Ten evaluation indicators were selected to represent the principal performance dimensions involved in the proposed LCRPB utilization pathway. Specifically, flowability was used to characterize fresh-state workability; 7- and 28-day compressive strengths and 7- and 28-day flexural strengths were used to represent early- and later-age mechanical performance; the time required to reach pH = 7 and the pH decline rate were used to describe the kinetic characteristics of wet carbonation; CO_2_ sequestration capacity and carbonation efficiency were used to characterize CO_2_ mineralization performance; and the post-carbonation activity index was used as an indicator of subsequent reuse potential. Collectively, these indicators cover engineering performance, carbonation behavior, CO_2_ sequestration, and post-carbonation recyclability, consistent with the overall objectives of this study. For each evaluation indicator, an individual weight vector $\omega_{q}$ (q = 1, 2, 3, …, 10) was obtained using the above matrix procedure. In the baseline multi-objective analysis, equal importance was assigned to the ten evaluation indicators. Equal weighting was adopted as a neutral reference scheme because no application-specific or independently validated preference structure was available to justify assigning greater priority to any particular indicator. This treatment does not imply that all indicators are intrinsically of identical practical importance; rather, it avoids introducing additional subjective assumptions into the baseline formulation selection. The robustness of this assumption was subsequently examined through a weighting sensitivity analysis. The comprehensive weight of each factor level was determined by averaging the ten individual weight vectors. The resulting comprehensive influence weights are listed in Table 16 and illustrated in Figure 22. Based on the calculated weights, A1B1C2D3E2, corresponding to W/B = 0.40, SF = 0%, GBFS = 7.5%, FA = 10%, and RCP = 10%, was identified as the preferred multi-objective formulation within the investigated factor space.

To evaluate the robustness of the formulation selection with respect to the weighting assumptions, a weighting sensitivity analysis was conducted using the equal-weight scheme as the baseline. Under the baseline scenario, each of the ten indicators was assigned a weight of 0.10, corresponding to aggregated weights of 0.50, 0.40, and 0.10 for engineering performance, carbon-sequestration performance, and post-carbonation recyclability, respectively. Three alternative weighting scenarios were further considered: an engineering-performance-oriented scenario (0.60/0.30/0.10), a carbon-sequestration-oriented scenario (0.30/0.60/0.10), and a recyclability-oriented scenario (0.40/0.30/0.30). As shown in Table 17, A1B1C2D3E2 remained the selected combination under both the engineering-performance-oriented and recyclability-oriented scenarios. Under the carbon-sequestration-oriented scenario, only the preferred water-to-binder ratio shifted from A1 (0.40) to A3 (0.50), whereas the preferred levels of SF, GBFS, FA, and RCP remained unchanged. These results indicate that the selection of SF, GBFS, FA, and RCP levels is relatively robust to variations in the weighting scheme, whereas the preferred W/B exhibits a certain degree of sensitivity when greater priority is assigned to carbon-sequestration performance. Overall, the limited variation in the selected factor levels suggests that the multi-objective formulation selection is not strongly dependent on a single weighting assumption. Considering the objective of achieving a balanced compromise among engineering performance, CO_2_ sequestration, and post-carbonation recyclability, A1B1C2D3E2 was therefore retained as the preferred multi-objective formulation within the investigated factor space.

Based on the multi-objective matrix analysis, A1B1C2D3E2 was identified as the preferred formulation within the investigated factor space. Notably, the same binder composition was also adopted in our previous recycled-concrete study [46], in which favorable workability, mechanical performance, and durability were experimentally demonstrated. The consistency between the present multi-objective selection and the previously observed engineering performance provides additional support for the rationality and practical applicability of the selected formulation.

### 3.11. Carbon Footprint and Carbon Reduction

According to the calculation method described in Section 2.5, the selected LCRPB formulation consisted of 72.5% OPC, 7.5% GBFS, 10% FA, and 10% RCP. Its material-related carbon footprint was estimated to be 0.670 kg CO_2_-eq/kg binder, compared with 0.912 kg CO_2_-eq/kg binder for the 100% OPC reference. Accordingly, the selected formulation achieved a 26.6% reduction in material-related carbon emissions relative to the OPC reference, mainly due to the partial replacement of OPC with supplementary and recycled constituents that have lower carbon emission factors.

Because the selected composition (A1B1C2D3E2) was identified through the multi-objective analysis and was not directly included as an individual mixture in the L25 orthogonal array, its CO_2_ sequestration capacity was estimated using the additive main-effect model. The predicted value was 20.043 g CO_2_/100 g hydrated powder. This value represents the CO_2_ sequestration response normalized to the mass of the 28-day hydrated powder used in the wet-carbonation test. Because this mass basis differs from the initial dry-binder basis adopted in the material-related carbon-footprint assessment, the CO_2_ sequestration capacity and material-related carbon footprint are reported as complementary but separate indicators of the low-carbon performance of LCRPB.

### 3.12. Limitations and Future Research

The findings of this study should be interpreted within the scope of the adopted experimental framework. First, the L25 orthogonal design was primarily intended to evaluate the main effects of mixture composition, while its ability to resolve factor interactions and experimental error is relatively limited. Future studies may employ expanded experimental designs to further examine interaction effects among RCP and supplementary cementitious materials. Second, the wet-carbonation tests were conducted under accelerated laboratory conditions using 100% CO_2_, and the calculated CO_2_ sequestration capacity was based mainly on crystalline CaCO_3_ retained in the analyzed solid fraction. Further studies under lower CO_2_ concentrations or representative flue-gas conditions, combined with more comprehensive carbon-balance measurements, would improve the assessment of practical carbonation performance. Third, the activity index used in this study provides a preliminary indication of post-carbonation reuse potential. Future work will further investigate workability, setting behavior, long-term mechanical properties, and durability of cement-based materials containing carbonated LCRPB. In addition, dedicated confirmation experiments for the formulation selected through multi-objective analysis will further evaluate the reliability and engineering applicability of the proposed optimization framework.

## 4. Conclusions

(1) The engineering performance of LCRPB is governed by the balance between constituent reactivity and the dilution associated with OPC replacement. Within the investigated ranges, W/B showed the greatest relative influence on flowability and 28-day compressive strength. At favorable dosage levels, 2% SF, 7.5% GBFS, and 10% FA increased the 28-day compressive strength by 17.43%, 12.43%, and 11.83%, respectively, whereas increasing the RCP content from 30% to 40% resulted in a 30.36% reduction in compressive strength. These results indicate that moderate incorporation of supplementary cementitious materials can improve performance, while excessive RCP replacement is dominated by dilution and reduced hydration.
(2) Wet carbonation demonstrated the CO_2_ sequestration potential of the multicomponent LCRPB system. Among the investigated carbonation conditions, a liquid-to-solid ratio of 5 and a CO_2_ flow rate of 3 L/min produced the most favorable response, with the carbonate-related mass loss of RCP reaching approximately 21.36%. The observed differences in pre-leaching behavior, pH evolution, and carbonation kinetics indicate that CO_2_ sequestration is governed by the combined effects of hydration-product composition, alkaline reserve, and the availability of carbonatable phases.
(3) The carbonated LCRPB powder retained appreciable residual activity when reused in cement-based mortar, demonstrating its potential for subsequent utilization after CO_2_ mineralization. The relatively stable activity-index response across the investigated compositions indicates that post-carbonation reuse performance is governed by the combined effects of the multicomponent binder system. These findings support the further utilization of carbonated LCRPB powder in cement-based materials and provide a basis for integrating CO_2_ sequestration with material recycling.
(4) Integrating engineering performance, carbonation behavior, CO_2_ sequestration, and post-carbonation activity through multi-objective matrix analysis identified a balanced formulation of W/B = 0.40, SF = 0%, GBFS = 7.5%, FA = 10%, and RCP = 10%. The selected formulation reduced the material-related carbon footprint by 26.6% relative to the 100% OPC reference. These results demonstrate that combining cement replacement, CO_2_ mineralization, and subsequent reuse provides a feasible pathway for the high-value and low-carbon utilization of recycled concrete powder.

## Figures and Tables

**Table 1:** Chemical Composition of Cementitious Materials (%).

Category/Ingredients	CaO	SiO_2_	Al_2_O_3_	Fe_2_O_3_	MgO	SO_3_	Na_2_O	K_2_O
OPC	63.21	18.48	6.74	3.45	3.24	3.16	0.17	0.53
FA	17.64	45.05	21.97	7.76	2.57	1.46	0.47	1.13
GBFS	39.69	32.34	17.67	1.98	3.26	1.35	2.80	3.22
SF	0.28	97.02	0.27	0.06	0.53	0.89	0.20	0.30
RCP	29.02	33.55	12.19	3.18	3.83	1.29	1.25	1.18

**Table 2:** Variables and Levels in the Orthogonal Experiment.

Level	Factors				
	A	B	C	D	E
	W/B	SF (%)	GBFS (%)	FA (%)	RCP (%)
K1	0.4	0	0	0	0
K2	0.45	2	7.5	5	10
K3	0.5	4	15	10	20
K4	0.55	6	22.5	15	30
K5	0.6	8	30	20	40

**Table 3:** Mix proportions for the orthogonal experiments (kg/m^3^).

Number	W/B	SF	GBFS	FA	RCP	OPC	Water
LCRPB1	0.5	0	0	0	0	1300	650
LCRPB2	0.5	26	195	195	520	364	650
LCRPB3	0.5	52	390	65	390	403	650
LCRPB4	0.5	78	97.5	260	260	604.5	650
LCRPB5	0.5	104	292.5	130	130	643.5	650
LCRPB6	0.45	0	390	195	260	455	585
LCRPB7	0.45	26	97.5	65	130	981.5	585
LCRPB8	0.45	52	292.5	260	0	695.5	585
LCRPB9	0.45	78	0	130	520	572	585
LCRPB10	0.45	104	195	0	390	611	585
LCRPB11	0.4	0	292.5	65	520	422.5	520
LCRPB12	0.4	26	0	260	390	624	520
LCRPB13	0.4	52	195	130	260	663	520
LCRPB14	0.4	78	390	0	130	702	520
LCRPB15	0.4	104	97.5	195	0	903.5	520
LCRPB16	0.55	0	195	260	130	715	715
LCRPB17	0.55	26	390	130	0	754	715
LCRPB18	0.55	52	97.5	0	520	630.5	715
LCRPB19	0.55	78	292.5	195	390	344.5	715
LCRPB20	0.55	104	0	65	260	871	715
LCRPB21	0.6	0	97.5	130	390	682.5	780
LCRPB22	0.6	26	292.5	0	260	721.5	780
LCRPB23	0.6	52	0	195	130	923	780
LCRPB24	0.6	78	195	65	0	962	780
LCRPB25	0.6	104	390	260	520	26	780

**Table 4:** Variables in the wet carbonation test.

Group	Liquid-to-Solid Ratio (L/S)	Group	CO_2_ Gas Flow Rate (L/min)
LSR1	4	GFR1	3
LSR2	5	GFR2	6
LSR3	6	GFR3	9

**Table 5:** Carbon emission factors used in the carbon footprint assessment.

Item	Emission Factor	Unit	Data Source/Basis
OPC	0.912	kg CO_2_-eq/kg	Almonayea et al. [43]
GBFS	0.0416	kg CO_2_-eq/kg	Almonayea et al. [43]
FA	0.0040	kg CO_2_-eq/kg	Almonayea et al. [43]
SF	0.0280	kg CO_2_-eq/kg	Almonayea et al. [43]
RCP	0.0488	kg CO_2_-eq/kg	Luo et al. [44]

**Table 6:** Analysis of Variance for Slurry Flowability in an Orthogonal Experiment.

Factors	SS	DF	MS	F	Significance
W/B	28,572.344	4	7143.086	52.398	0.001
SF	5966.561	4	1491.640	10.942	0.02
GBFS	698.407	4	174.602	1.281	0.408
FA	2598.940	4	649.735	4.766	0.08
RCP	1931.402	4	482.851	3.542	0.124
Error	545.292	4	136.323		

**Table 7:** Analysis of Variance for the 28-Day Compressive Strength of Cement Paste in an Orthogonal Experiment.

Factors	SS	DF	MS	F	Significance
A	770.727	4	192.682	6.638	0.047
B	56.318	4	14.079	0.485	0.750
C	102.530	4	25.633	0.883	0.547
D	78.601	4	19.650	0.677	0.643
E	678.575	4	169.644	5.844	0.058
Error	116.109	4	29.027		

**Table 8:** Analysis of Variance for the 28-Day Flexural Strength of Cement Paste in an Orthogonal Experiment.

Factors	SS	DF	MS	F	Significance
A	64.399	4	16.100	26.730	0.004
B	13.135	4	3.284	5.452	0.065
C	16.245	4	4.061	6.743	0.046
D	8.327	4	2.082	3.456	0.128
E	90.557	4	22.639	37.588	0.002
Error	2.409	4	0.602		

**Table 9:** Weight Loss of Calcium Carbonate in Thermogravimetric Analysis at Different Liquid-to-Solid Ratios and Gas Flow Rates.

liquid-to-Solid Ratio (L/S)	Loss in Weight of Calcium Carbonate (%)	Gas Flow Rate (L/min)	Loss in Weight of Calcium Carbonate (%)
4	20.25943	3	21.35893
5	21.35893	6	20.66796
6	20.47458	9	15.09943

**Table 10:** Analysis of Variance of the LCRPB’s Carbon Sequestration Capacity.

Factors	SS	DF	MS	F	Significance
A	78.826	4	19.707	0.296	0.867
B	187.102	4	46.776	0.702	0.630
C	54.201	4	13.550	0.203	0.924
D	310.188	4	77.547	1.164	0.443
E	164.647	4	41.162	0.618	0.674
Error	266.475	4	66.619		

**Table 11:** Analysis of variance for the time from pre-leaching to stabilization.

Factors	SS	DF	MS	F	Significance
A	162.640	4	40.660	2.239	0.227
B	61.440	4	15.360	0.846	0.563
C	47.440	4	11.860	0.653	0.655
D	48.640	4	12.160	0.670	0.646
E	17.040	4	4.260	0.235	0.905
Error	72.640	4	18.160		

**Table 12:** Analysis of variance for the stable pH value during pre-leaching.

Factors	SS	DF	MS	F	Significance
A	1.616	4	0.404	0.451	0.770
B	1.295	4	0.324	0.362	0.826
C	0.377	4	0.094	0.105	0.975
D	1.829	4	0.457	0.511	0.734
E	1.156	4	0.289	0.323	0.851
Error	3.582	4	0.896		

**Table 13:** Analysis of variance for the rate of pH decline during the carbonation process.

Factors	SS	DF	MS	F	Significance
A	0.109	4	0.027	0.862	0.556
B	0.117	4	0.029	0.924	0.529
C	0.302	4	0.075	2.390	0.210
D	0.067	4	0.017	0.527	0.725
E	0.464	4	0.116	3.673	0.118
Error	0.126	4	0.032		

**Table 14:** Analysis of variance for the time required for carbonation to pH =7.

Factors	SS	DF	MS	F	Significance
A	450.400	4	112.600	6.086	0.054
B	106.400	4	26.600	1.438	0.367
C	323.600	4	80.900	4.373	0.091
D	12.800	4	3.200	0.173	0.941
E	590.800	4	147.700	7.984	0.034
Error	74.000	4	18.500		

**Table 15:** Analysis of Variance for the Activity Index of Cementitious Materials After Hydration and Carbonation.

Factors	SS	DF	MS	F	Significance
A	0.147	4	0.037	1.744	0.302
B	0.009	4	0.002	0.109	0.973
C	0.015	4	0.004	0.177	0.939
D	0.032	4	0.008	0.377	0.816
E	0.022	4	0.006	0.263	0.888
Error	0.084	4	0.021		

**Table 16:** Comprehensive influence weights of each factor level.

Factor	Level	Comprehensive Weight
A (W/B)	0.4	0.0643
	0.45	0.0622
	0.5	0.0616
	0.55	0.0564
	0.6	0.0544
B (SF content (%))	0	0.0341
	2	0.0300
	4	0.0304
	6	0.0289
	8	0.0292
C (GBFS content (%))	0	0.0294
	7.5	0.0342
	15	0.0283
	22.5	0.0276
	30	0.0234
D (FA content (%))	0	0.0362
	5	0.0331
	10	0.0378
	15	0.0323
	20	0.0324
E (RCP content (%))	0	0.0486
	10	0.0563
	20	0.0526
	30	0.0421
	40	0.0342

**Table 17:** Sensitivity of the selected LCRPB formulation to different indicator-weighting schemes.

Indicator Category	Evaluation Indicator	Equal-Weight Baseline	Engineering-Performance-Oriented	Carbon-Sequestration-Oriented	Recyclability-Oriented
Engineering performance	Flowability	0.100	0.120	0.060	0.080
	7-day compressive strength	0.100	0.120	0.060	0.080
	7-day flexural strength	0.100	0.120	0.060	0.080
	28-day compressive strength	0.100	0.120	0.060	0.080
	28-day flexural strength	0.100	0.120	0.060	0.080
Carbon-sequestration performance	Time required to reach pH = 7	0.100	0.075	0.150	0.075
	pH decline rate	0.100	0.075	0.150	0.075
	CO_2_ sequestration capacity	0.100	0.075	0.150	0.075
	Carbonation efficiency	0.100	0.075	0.150	0.075
Post-carbonation recyclability	Post-carbonation activity index	0.100	0.100	0.100	0.300
Total		1.000	1.000	1.000	1.000
Selected combination		A1B1C2D3E2	A1B1C2D3E2	A3B1C2D3E2	A1B1C2D3E2
Indicator category	Evaluation indicator	Equal-weight baseline	Engineering-performance-oriented	Carbon-sequestration-oriented	Recyclability-oriented

## Data Availability

The original contributions presented in this study are included in the article. Further inquiries can be directed to the corresponding authors.
